# One-month spaceflight compromises the bone microstructure, tissue-level mechanical properties, osteocyte survival and lacunae volume in mature mice skeletons

**DOI:** 10.1038/s41598-017-03014-2

**Published:** 2017-06-01

**Authors:** Maude Gerbaix, Vasily Gnyubkin, Delphine Farlay, Cécile Olivier, Patrick Ammann, Guillaume Courbon, Norbert Laroche, Rachel Genthial, Hélène Follet, Françoise Peyrin, Boris Shenkman, Guillemette Gauquelin-Koch, Laurence Vico

**Affiliations:** 1 French National Centre for Space Studies, Paris, France; 2 INSERM, UMR 1059, University of Lyon, University Jean Monnet, F42023 Saint-Etienne, France; 3 INSERM, UMR 1033, University of Lyon, University Claude Bernard Lyon 1, F69622 Lyon, France; 4 University of Lyon, INSERM U1206, France and European Synchrotron Radiation Facility, CS40220, 38043 Grenoble Cedex 9, France; 5 Division of Bone Diseases, Department of Internal Medicine Specialties, Geneva University Hospitals and Faculty of Medicine, Geneva, Switzerland; 6 CNRS UMR 5588, University of Grenoble Alpes, Grenoble, France; 7 Institute for Biomedical Problems, Russian Academy of Sciences, Moscow, Russia

## Abstract

The weightless environment during spaceflight induces site-specific bone loss. The 30-day Bion-M1 mission offered a unique opportunity to characterize the skeletal changes after spaceflight and an 8-day recovery period in mature male C57/BL6 mice. In the femur metaphysis, spaceflight decreased the trabecular bone volume (−64% vs. Habitat Control), dramatically increased the bone resorption (+140% vs. Habitat Control) and induced marrow adiposity invasion. At the diaphysis, cortical thinning associated with periosteal resorption was observed. In the Flight animal group, the osteocyte lacunae displayed a reduced volume and a more spherical shape (synchrotron radiation analyses), and empty lacunae were highly increased (+344% vs. Habitat Control). Tissue-level mechanical cortical properties (i.e., hardness and modulus) were locally decreased by spaceflight, whereas the mineral characteristics and collagen maturity were unaffected. In the vertebrae, spaceflight decreased the overall bone volume and altered the modulus in the periphery of the trabecular struts. Despite normalized osteoclastic activity and an increased osteoblast number, bone recovery was not observed 8 days after landing. In conclusion, spaceflight induces osteocyte death, which may trigger bone resorption and result in bone mass and microstructural deterioration. Moreover, osteocyte cell death, lacunae mineralization and fatty marrow, which are hallmarks of ageing, may impede tissue maintenance and repair.

## Introduction

Spaceflight sojourns induce a series of physiological adaptations in the human body that predispose astronauts to an increased risk of bone fracture after returning to Earth. Under the weightless spaceflight environment, astronauts are subject to factors such as radiation exposure, biorhythm changes, and vestibular alterations, and spaceflight induces site-specific bone loss in both humans and rodents^1–5^.

Previous in-flight animal research conducted on young growing rats during relatively short-term missions (from 4 to 19 days) has considerably advanced our understanding of skeletal adaptations to spaceflight, including the mechanisms of bone loss and skeletal development in microgravity^6^. These experiments showed either deterioration in trabecular and cortical bone parameters^7–11^ or no changes^12–16^ depending on the animal’s age, strain levels, habitat, flight duration and the delay between landing and sample collection. Nevertheless, in the context of growing bone, most of the results provide evidence for the spaceflight-induced inhibition of bone formation as well as impairment in periosteal expansion and longitudinal growth^7–11^, whereas bone resorption is either unchanged^17,18^ or increased^2^. Impaired mechanical properties have also been reported in the humerus and vertebrae^11,19^, although these changes were mostly caused by geometrical characteristics rather than differences in material properties^19^. Studies have also provided evidence of alterations in the bone matrix, mineral maturation and degree of mineralization (DMB) in the cortical compartment, suggesting that spaceflight also affects bone cortical-quality properties in growing rats^2,20–23^.

Because of the relationship between launch weight and cost as well as the constraints of launch vehicles, lighter experiments that use mice are now more common. The availability of multiple genetically modified mice strains also offers additional research opportunities. Excluding the topic of this paper (the Bion M1 flight), four previous mice missions have provided data on in-flight skeletal changes. These missions included short 12–15 day shuttle missions (STS-108, 131 and 135) with female C57BL6/J mice (2 to 4.5 months old) and a 91-day mission aboard the International Space Station (ISS) with 2-month-old male mice. Unfortunately, in the latter experiment, only 1 wild-type and 2 pleiotrophin-transgenic mice survived the flight^24^. In brief, investigations of the tibia, femur and caudal vertebrae^24–26^ or the ischium^1^ revealed bone loss with increased bone resorption^1,26^ and decreased bone formation^24,26^.

Taken together, data from rats and mice emphasize an imbalance between bone resorption and formation, which results in deep alterations of the bone structure under space conditions. Furthermore, the bone loss appears to increase with time as also observed in space crewmembers^3^, highlighting the usefulness of prolonged mission studies. In addition, bone recovery after spaceflight is a concern because studies on spacemen^3,27^ and young rats^2,28^ have suggested that bone mass recovery after reambulation takes longer than the mission duration.

Thus, our knowledge of skeletal changes during and after spaceflight remains incomplete. The BionM1 mission provided additional invaluable evidence to help further our understanding of the cellular, microstructural and qualitative bone changes that occur in skeletally mature mice^29,30^ after a one-month mission. In addition, a subset of flight mice was allowed to recover over the 8 days after landing to study their behavioural and locomotor functions^31^. Overall, studying the early dynamic events during bone re-adaptation to normal gravity is important, particularly for the development and prescription of post-flight countermeasures.

Therefore, we analysed the microstructure of trabecular and cortical bone in the peripheral and axial skeleton of mature male mice and hypothesized that greater bone loss would be observed in the weight-bearing femur compared with the less weight-bearing vertebra. We also performed a cellular analysis of osteoblasts, osteoclasts, osteocytes and adipocytes at the different sites. Finally, the bone sub-microstructure and nanostructure were investigated to assess bone fragility, bone matrix collagen and mineralization properties as well as the 3D osteocyte-lacuna morphometric shape and distribution.

## Results

Four groups of C57BL/6N mature male mice (23 weeks old) were compared. Two groups were exposed to a one-month spaceflight: (i) a Flight group, which included mice killed within the day after landing; and (ii) a Flight + Rec recovery group, which included mice that were allowed to recover over 8 days. Two ground control groups were also included: a Habitat Control group, which was kept under spacecraft housing conditions; and a Control group, which was kept under standard housing conditions (for more details, see the Methods section).

### Effects of spaceflight, Earth recovery and spacecraft housing conditions on body mass and longitudinal growth

**Figure 1:**
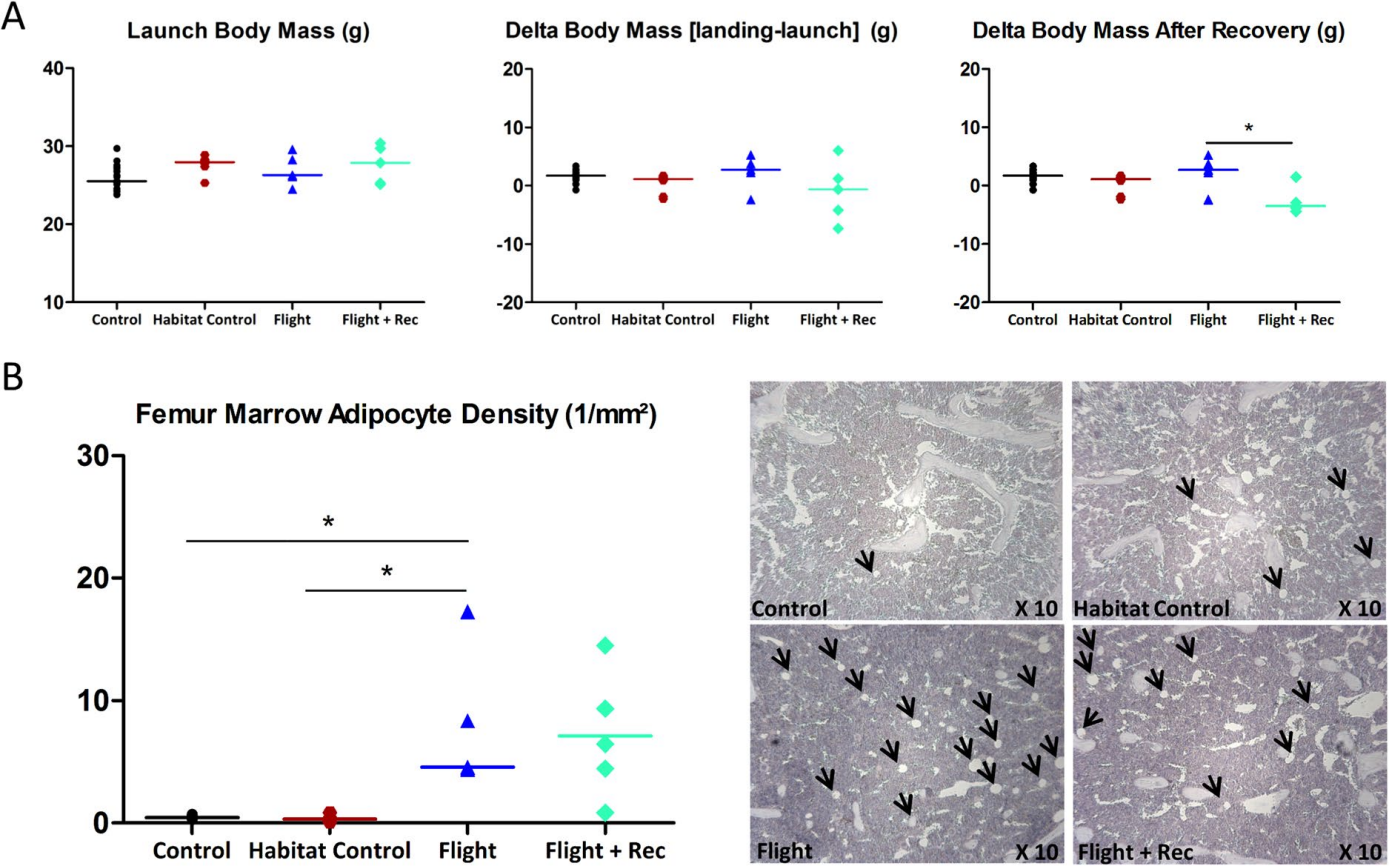
Effect of spacecraft housing conditions, spaceflight and recovery on (**A**) body mass, and (**B**) femur marrow adipocyte density (black arrows) in the metaphysis trabecular compartment (representative Haematoxylin/Eosin stained longitudinal sections). Data are presented as clusters of individual points (bars = median). *p < 0.017, **p < 0.003.

The body mass did not differ between the groups before the flight and at landing (Fig. 1A and Table S1). During the recovery period, the animal body mass decreased in the Flight + Rec group (−2.6 ± 1.1 g; p < 0.017 vs. Flight) (Fig. 1A and Table S1). Histological measurements indicated that the growth plate was significantly thinner in the Habitat Control vs. Control groups (p < 0.017) (Table S1), whereas significant differences were not observed between the other groups. In addition, in all groups, primary spongiosae were absent and femur length alterations were not observed, thus highlighting the cessation of longitudinal growth (Table S1).

### Effects of spacecraft housing conditions on vertebrae and femurs

**Figure 2:**
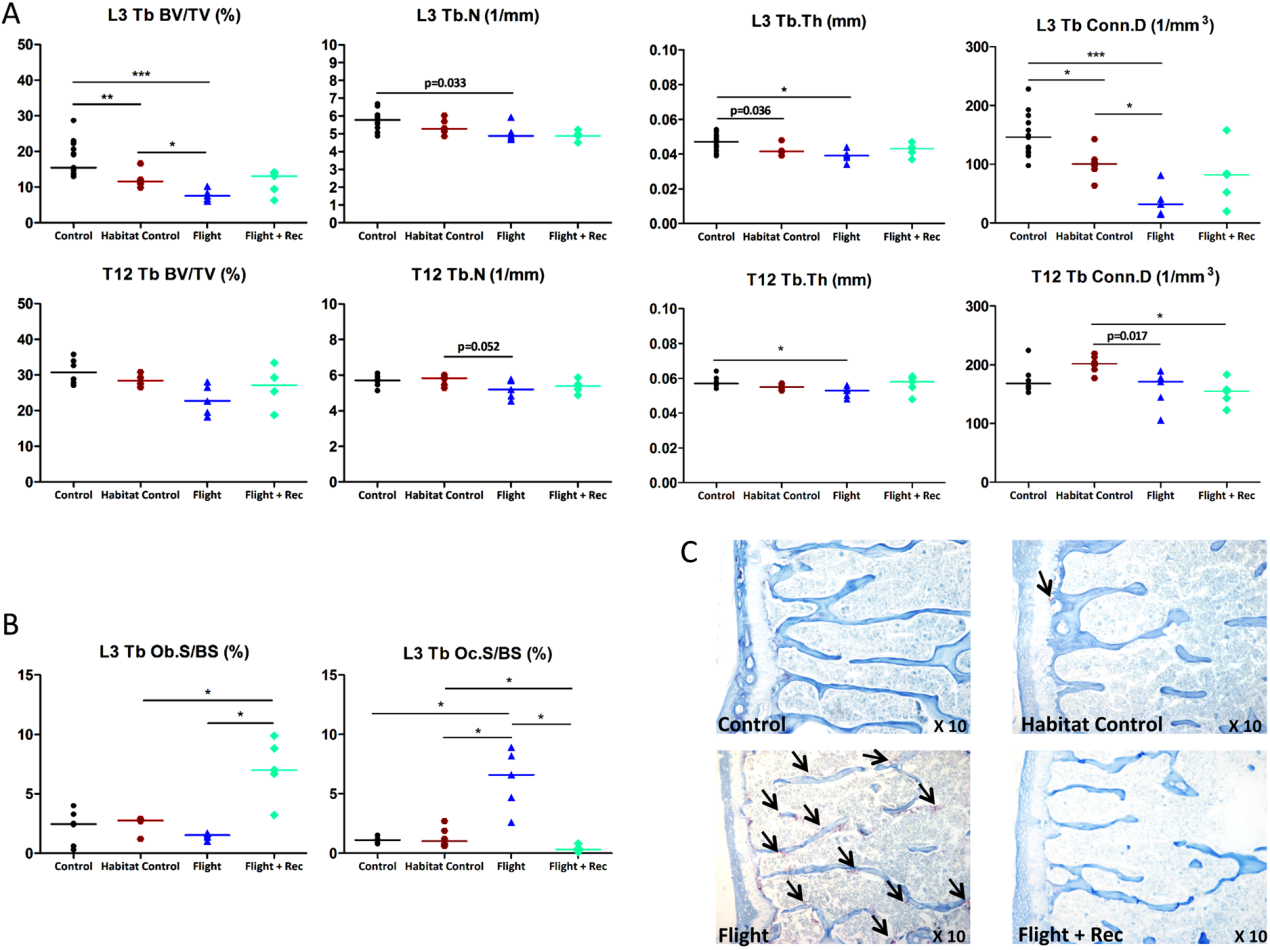
Effects of spacecraft housing conditions, spaceflight and recovery on (**A**) L3 and T12 vertebrae microarchitecture parameters, (**B**) trabecular osteoblast surfaces (Ob.S/BS) and osteoclast surfaces (Oc.S/BS). (**C**) Tartrate resistant acid phosphatase staining of osteoclastic cells (black arrows). Trabecular (Tb) parameters include bone volume fraction (BV/TV), trabecular number (Tb.N), trabecular thickness (Tb.Th), and connectivity density (Conn.D). Data are presented as clusters of individual points (bars = median). *p < 0.017, **p < 0.003.

**Figure 3:**
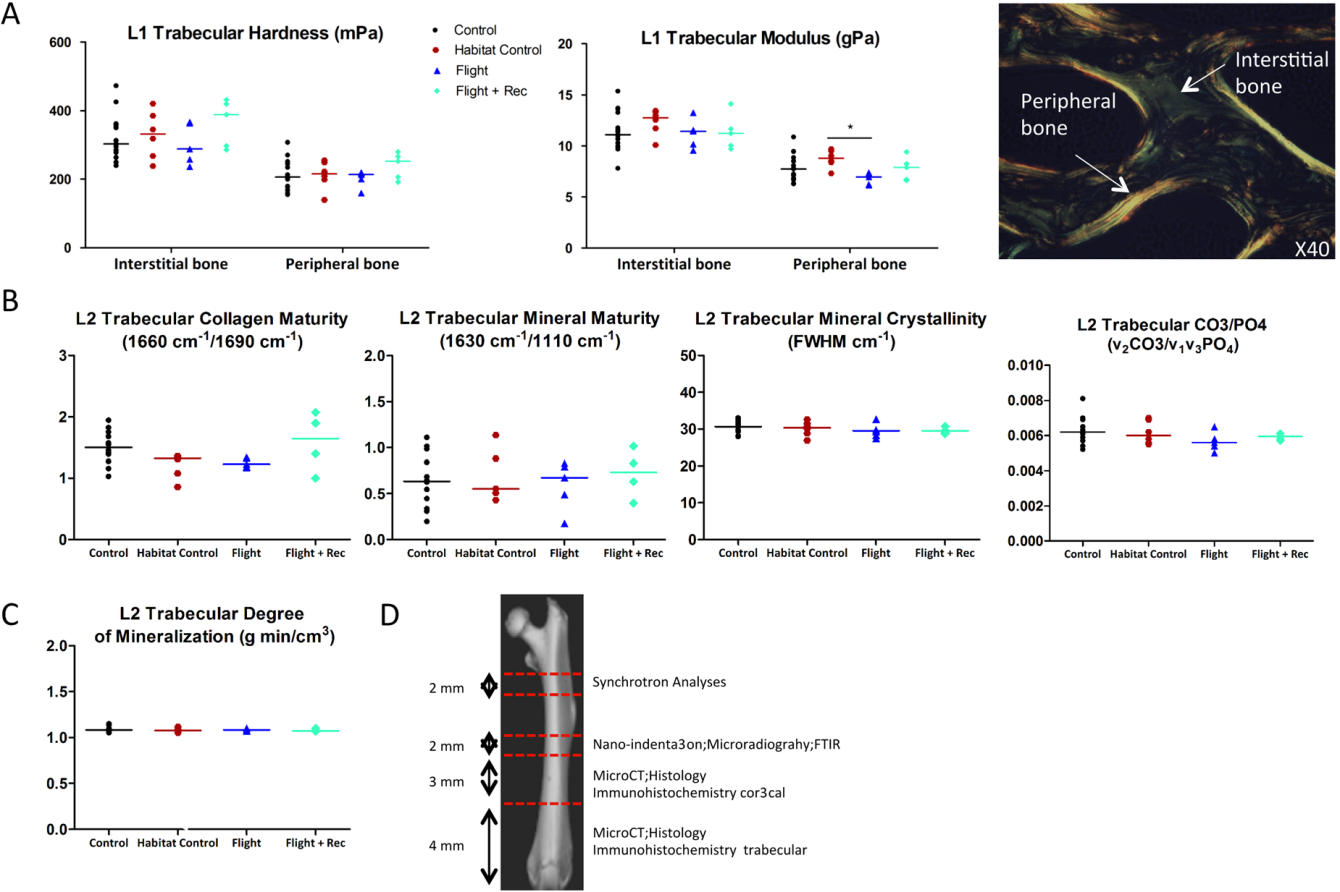
Effects of spacecraft housing conditions, spaceflight and recovery on (**A**) L1 vertebra biomechanical properties. The polarized light image on the right illustrates the two regions of interest for biomechanical analyses within vertebra trabeculae (**B**) L2 FTIRM parameters and (**C**) L2 DMB. Data are presented as clusters of individual points (bars = median). *p < 0.017, **p < 0.003. (**D**) Representative schema of the investigated zones in femur.

**Figure 4:**
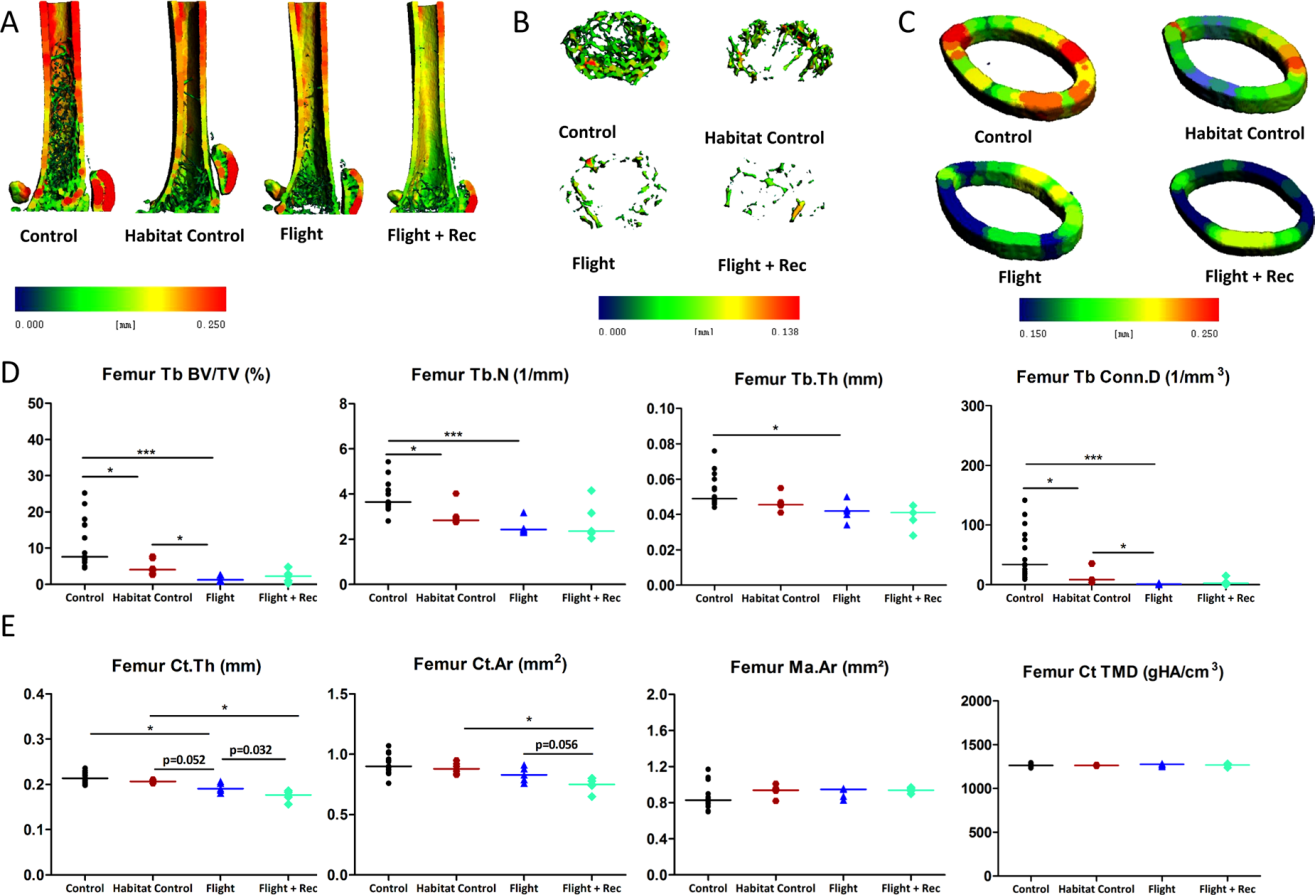
Effect of spacecraft housing conditions, spaceflight and recovery on (**A**) the femur total microarchitecture, (**B**) the metaphysis trabecular (Tb) microarchitecture, and (**C**) the cortex at mid-diaphysis. A panel thickness colour map of representative 3D reconstructions illustrates the decrease in bone mass, Tb volume and cortical (Ct) thickness induced by microgravity and after Earth recovery. (**D**) Tb microarchitecture parameters and (**E**) Ct parameters. Tb parameters include bone volume fraction (BV/TV), trabecular number (Tb.N), trabecular thickness (Tb.Th), and connectivity density (Conn.D). Ct bone parameters include Ct thickness (Ct.Th), Ct area (Ct.Ar), tissue mineral density (TMD), and marrow area (Ma.Ar). Data are presented as clusters of individual points (bars = median). *p < 0.017, **p < 0.003.

**Figure 5:**
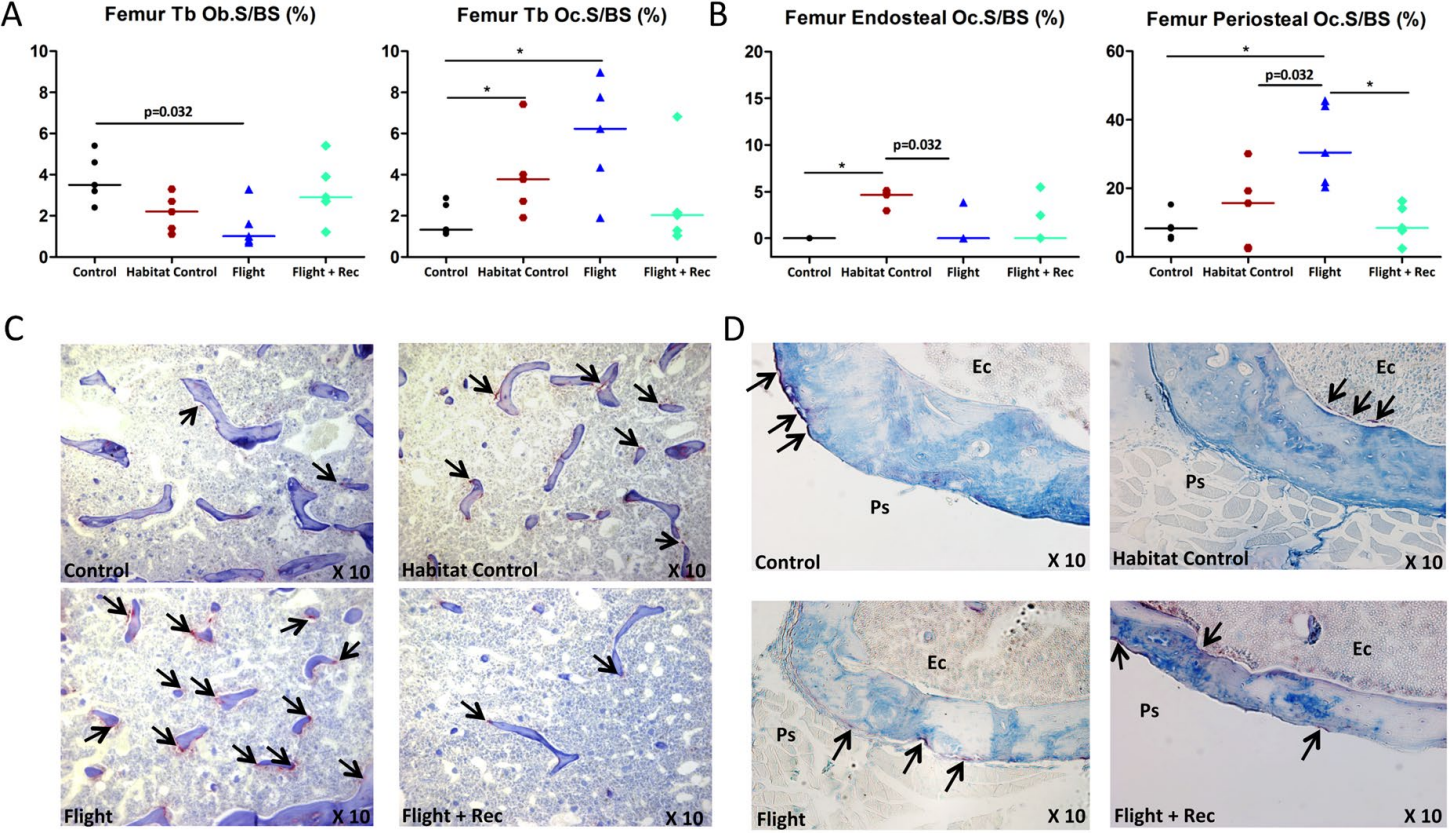
Effect of spacecraft housing conditions, spaceflight and recovery on (**A**) femur trabecular osteoblast surfaces (Ob.S/BS) and osteoclast surfaces (Oc.S/BS), and (**B**) cortical osteoclast surfaces. Tartrate resistant acid phosphatase staining of osteoclastic cells (black arrows) in the trabecular compartment (**C**), in the endocortical surface (Ec) and in the periosteal surface (Ps) (**D**). Data are presented as clusters of individual points (bars = median). p < 0.017, **p < 0.003.

**Figure 6:**
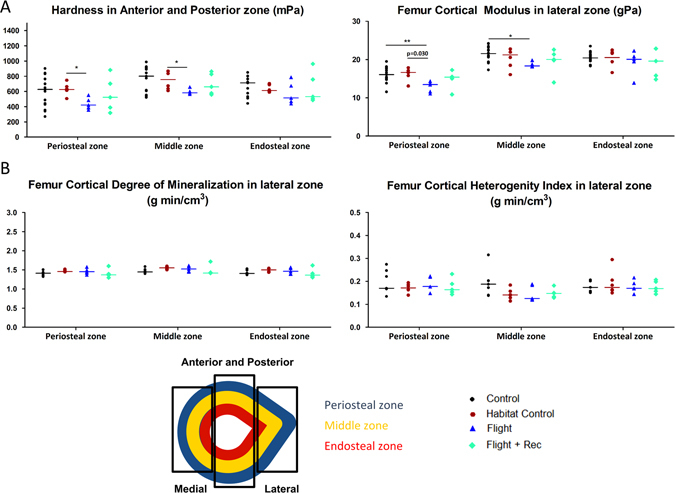
Effects of spacecraft housing conditions, spaceflight and recovery on (**A**) femur cortical biomechanical properties, (**B**) femur cortical mineralization parameters in the lateral zone (schema in the upper right corner illustrates the regions of analysis), and (**C**) cortical FTIRM parameters. Data are presented as clusters of individual points (bars = median). *p < 0.017, **p < 0.003.

**Figure 7:**
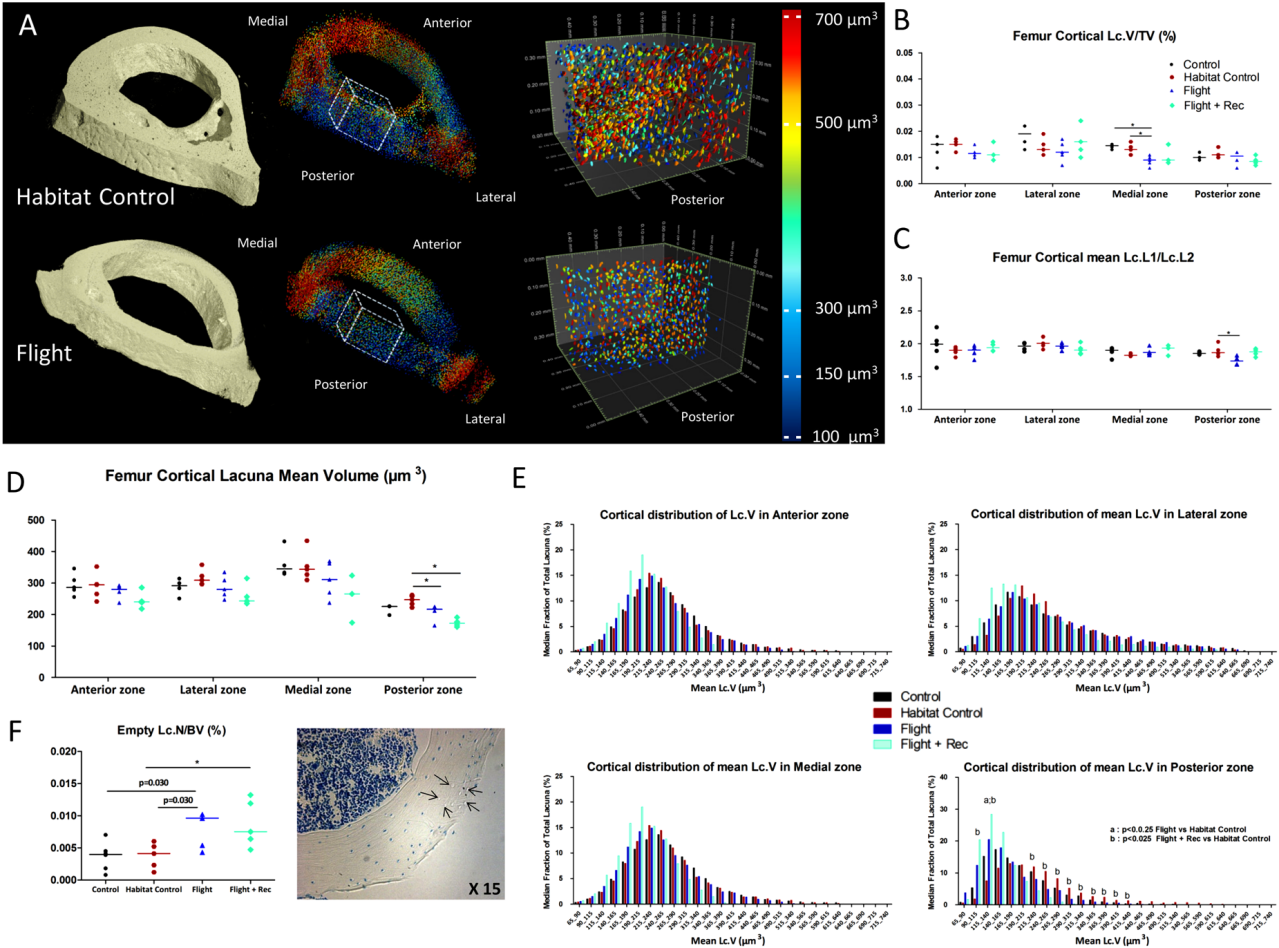
Effects of spacecraft housing conditions, spaceflight and recovery on osteocyte lacunae volume and shape. (**A**) A 3D colour map reconstruction of synchrotron radiation imaging showing total bone, total lacunae, and magnification of posterior zones in one Flight and Habitat Control mouse. (**B**,**C**) Lacunae total volume and shape (Lc.L1/Lc.L2), (**D**,**E**) lacunae mean volume and distribution, and (**F**) empty lacunae fraction measured by histomorphometry. Data are presented as clusters of individual points (bars = median). p < 0.017, **p < 0.003.

A comparison between the two control groups (Control and Habitat Control) was performed to estimate the impact of spacecraft housing, which was found to negatively affect the femur and lumbar vertebrae (but not the thoracic vertebrae). Indeed, the L3 vertebrae and femur trabecular bone volume fraction (BV/TV) and connectivity density were decreased in the Habitat Control group vs. the Control group (Figs 2A and 4A,B,D; and Table S2). After scanning, the femur length was assessed with a digital calliper and then cut with a precision diamond wire saw (Well, Escil, Chassieu, France) to allow for several analyses as shown in Fig. 3D. In the femur, the decreased BV/TV was related to fewer trabeculae (Fig. 4B,D; and Table S2) and increased osteoclast surfaces (Fig. 5A,C; and Table S3). However, the bone cellular parameters remained unchanged in the L3 vertebrae (Fig. 2B and Table S3). The femur cortical thickness and bone marrow area were unaltered despite a significant increase in endosteal resorption (Figs 4E and 5B; and Tables S2, S3). Spacecraft housing conditions did not alter the bone material properties as assessed by nanoindentation (Fig. 6A and Table S6); the matrix characteristics as assessed by Fourier transform infrared spectroscopy (FTIR) (Table S4); and the osteocyte lacunae shape, size and volume as assessed by synchrotron radiation (SR) imaging (Fig. 7, Table S7).

### Effects of spaceflight and Earth recovery on the vertebrae

The trabecular BV/TV of the L3 vertebrae analysed by microtomography decreased in the Flight group vs. both the Habitat Control and Control groups (−35.7% and −56.5%, respectively; p < 0.0033) because of decreases in the trabecular number (Tb.N) and thickness (Tb.Th) (Fig. 2A and Table S2). These parameters initiated a recovery in the Flight + Rec group (Fig. 2A and Table S2). In the T12 vertebrae, which were subjected to respiratory cage movements, the microarchitecture displayed milder deterioration than that of the L3 in the Flight group (Fig. 2A and Table S2). The L3 osteoclastic surfaces were dramatically increased in the Flight group vs. the Control, Habitat Control and Flight + Rec groups (+564%, +470% and +3836%, respectively; p < 0.017) (Fig. 2B,C and Table S3), which likely resulted in the decreased connectivity density in the Flight group (Fig. 2A and Table S2). The L3 osteoblastic surfaces were unaffected in the Flight group but increased in the Flight + Rec group vs. the Flight and Habitat Control groups (+450% and + 270%, respectively; p < 0.025) (Fig. 2B and Table S3). A bone formation activity rebound in the Flight + Rec group was also observed in the L1 vertebrae, where the osteoid surfaces (OS/BS, %) were increased vs. the Flight and Habitat Control groups (2.9 ± 0.4, 1.6 ± 0.2, and 1.5 ± 0.1, respectively; p < 0.017) (Table S3). Notably, the trabecular structural changes were not associated with trabecular tissue mineral density alterations in both lumbar vertebrae (Table S2).

To evaluate whether spaceflight and recovery also altered the bone material properties, nanoindentation analyses of the L1 interstitial (older) and peripheral trabecular bone (younger bone) were performed (see the image in Fig. 3A). At the interstitial level, the hardness, maximal force, dissipated energy, and modulus were not different between the groups (Fig. 3A and Table S6). At the peripheral level, the modulus was decreased in the Flight vs. Habitat Control group (−22%; p < 0.017) (Fig. 3A and Table S6). Because the elastic properties of the lumbar trabeculae were affected by spaceflight, we further investigated the intrinsic quality properties of collagen and mineral by FTIR spectroscopic microscopy (FTIRM) as well as the DMB by microradiography in the L2 vertebrae. The collagen maturity, mineral maturity, mineral crystallinity and carbonation as well as the DMB of the L2 vertebrae remained unchanged between the groups (Fig. 3B,C; and Tables S4, S5).

### Effects of spaceflight and Earth recovery on the femur

Because we hypothesized that weight-bearing sites would be more prone to spaceflight-induced bone loss compared with the vertebrae, the femur was further investigated with respect to the metaphyseal trabecular and diaphyseal cortical compartments. Figure 4 illustrates the microarchitecture alterations in the proximal mid-femur (A), the trabecular metaphyseal region (B) and the mid-diaphyseal cortical region (C). The trabecular BV/TV showed a large reduction in the Flight group vs. the Control and Habitat Control groups (−85.2%, p < 0.0003; −64.8%, p < 0.017; respectively) (Fig. 4D and Table S2). Similar trabecular parameters were found in the Flight + Rec and Flight groups, indicating that bone structure recovery was not initiated one week into the recovery period (Fig. 4D and Table S2). The trabecular osteoblastic surfaces tended to be decreased in the Flight vs. Control groups, although the changes were not significant (p = 0.032) (Fig. 5A and Table S3). A nonsignificant increase in the osteoblastic surfaces in the Flight + Rec group vs. the Flight group was also observed (Fig. 5A and Table S3). Trabecular osteoclastic surfaces were dramatically increased in the Flight group vs. the Control and Habitat Control groups (+337% and +148%, respectively; p < 0.017) (Fig. 5A,C; and Table S3), whereas they were decreased in the Flight + Rec group, although the changes were not significant (p = 0.177). Femur bone marrow histological analyses revealed that marrow adipocytes numbered close to zero in the Control and Habitat Control groups and were greatly increased in the Flight and Flight + Rec groups (p < 0.017) (Fig. 1B and Table S3).

The femur cortical thickness tended to decrease between the Flight and Habitat Control (p = 0.052) groups and between the Flight + Rec and Flight groups (p = 0.032), and it was significantly decreased between the Flight and Control groups and between the Flight + Rec and Habitat Control groups (p < 0.017; Fig. 4C,E; and Table S2). The femur cortical bone area presented a similar profile (Fig. 4E and Table S2). Cortical thinning without marrow area changes suggests periosteal resorption. Thus, we investigated osteoclastic cells at both the periosteal and endosteal levels. Periosteal osteoclastic surfaces were indeed increased in the Flight group vs. the Control and Flight + Rec groups and tended to increase in the Flight group vs. the Habitat Control group (Fig. 5B,D and Table S3). However, at the endosteum, osteoclastic surfaces did not display any changes in the Flight and Flight + Rec groups (Fig. 5B,D; and Table S3). As observed in the vertebrae, the femur trabecular and cortical tissue mineral densities were unaffected by the experimental conditions (Table S2).

The bone material properties of the femur cortex were locally analysed using nanoindentation in the medial, lateral, anterior and posterior quadrants of the transversal mid-diaphyses sections. In each quadrant, six indents per layer (periosteal, middle and endosteal) were performed (see the schema in Fig. 6). A significant decrease in hardness was observed in the Flight group vs. the Habitat Control group in the anterior and posterior zones at the periosteal and middle levels (Fig. 6A and Table S6). The modulus was also altered in the lateral periosteal and middle zones (Table S6). In other zones, the biomechanical properties of the femur did not differ between the groups (Table S6). A microradiography analysis of the femur cortex did not reveal any differences in the degree or heterogeneity of mineralization in the analysed zones (Fig. 6B and Table S5). Moreover, infrared spectroscopy analyses did not reveal any differences in the collagen or mineral quality properties between the groups throughout the entire cortical ring (Table S4).

To investigate whether spaceflight and/or Earth recovery affects the osteocyte lacunae morphology, femur cortical lacunae were investigated using SR nanoCT imaging. Four zones (lateral, medial, anterior and posterior) containing more than 1500 lacunae each were assessed. Figure 7A shows the total cortical ring 3D reconstruction, the total lacunae inside the ring and a magnification of the posterior zone. The lacunar volume fraction was significantly decreased in the Flight group vs. the Habitat Control and Control groups in the medial zone (−33% and −38%, respectively; p < 0.017) (Fig. 7B and Table S7). In the posterior zone, the length (L1)/width (L2) ratio was significantly decreased in the Flight group vs. the Habitat Control group, indicating a more spherical lacuna shape in the Flight mice (Fig. 7C and Table S7). Moreover, the lacuna mean volume was also significantly decreased in the Flight group vs. the Habitat Control group and in the Flight + Rec group vs. the Habitat Control group, particularly in the posterior zone (Fig. 7D and Table S7). In other zones, the mean volume of lacunae showed decreases, although the changes were not significant. Regarding the lacunae volume distribution (Fig. 7E), the fraction of smaller lacunae (less than 200 µm^3^) was greater in the Flight and Flight + Rec groups vs. the Habitat Control and Control groups and the fraction of larger lacunae (more than 200 µm^3^) was smaller in the Flight and Flight + Rec groups vs. the control groups (Fig. 7E).

To determine whether osteocyte survival was compromised by spaceflight, we assessed the number of empty lacunae via a histological analysis (Fig. 7F). All lacunae per cortical slices were investigated (Control, n = 443 ± 23; Habitat Control, n = 495 ± 49; Flight, n = 375 ± 64; Flight + Rec, n = 392 ± 69; Table S3). These investigations showed that the ratio of empty to total lacunae was dramatically increased in the Flight group vs. the Habitat Control and Control groups (11.4%, 4.6%, and 3.6%, respectively; p < 0.017) and remained elevated in the Flight + Rec group (9.7%) (Fig. 7F and Table S3).

## Discussion

This study provides multilevel analyses of weight-bearing (femur) and less weight-bearing (vertebrae) bones of the mature mouse skeleton after a one-month residency in space without any countermeasures and with an 8-day recovery period in male mice. We first confirmed the site-specific cortical and trabecular alteration induced by spaceflight, the weight bearing femur being more severe affected than vertebra^2,5^. We further revealed a number of unidentified spaceflight-induced effects. The nanomechanical characterization showed that the intrinsic mechanical properties were locally affected in both the axial and appendicular skeletal sites. We also demonstrated that spaceflight compromised the cortical osteocyte survival and decreased the lacunar volume. Furthermore, 8 days after landing, these bone adaptations did not recover despite normalized bone resorption and increased bone formation.

Although the small sample size of the mice on board Bion-M1 (n = 5 for the Flight and Flight + Rec groups) and the inter-individual variability limited the statistical power of our analyses, a nonparametric test with a p-value < 0.017, which was considered significant (Bonferroni’s adjustment), provided robustness by limiting the risk of false interpretation.

Before specifying the spaceflight effects, we acknowledge that spacecraft housing, per se, as observed in the Habitat Control group induced alterations compared with the standard Control group. Indeed, deteriorated trabecular bone was observed in the femur and vertebrae, although the changes were less severe than those in the Flight group. Compared with the Flight group, the cortical bone structure, material properties and osteocyte parameters in the Habitat Control group were preserved, even when endosteal bone resorption was specifically increased in this group. The spacecraft housing (Habitat Control, Flight and Flight + Rec) consisted of a cylindrical module, and it primarily differed from the standard housing (Control) by the space volume. Such confinement and the resulting reduction in physical activity likely induced the observed bone alterations. Furthermore, a special paste-like diet and distribution (6 times daily) may have contributed to the changes, although the nutrient and energy intake was generally consistent between groups^31^ and body mass changes were not observed among the Control, Habitat Control and Flight animals. The effects of a JAXA habitat mouse cage (different from the Bion-M1) have been tested in young mice^32^, and contrary to our results, femur cortical thinning without alteration of the trabecular compartment was observed. These observations highlight the importance of dissociating the effects of specific spacecraft housing conditions from spaceflight effects when interpreting results. Noteworthy, however, the mice experienced different spatial environments in the spacecraft housing on Earth (2D surface) and in flight (entire cylindrical volume).

In mice of the spaceflight groups, bone resorption was dramatically stimulated in both the trabecular femur and vertebrae, which is consistent with reports in shorter^1,26^ or longer^24^ missions with mice. Moreover, spaceflight-induced fat marrow invasion at the femur metaphysis based on a significant appearance of lipid droplets that remained 8 days post flight. This in-flight fat increase was previously reported in the humeral bone marrow of young growing rats^17^ as well as in human vertebrae after 60 days of bed rest^33^. At this stage, linking adipogenesis with a potential decrease in osteoblastogenesis is still speculative, although these lineages originate from the same precursor cells. The osteoblastic activity (posterior to osteoblastogenesis) was assessed by quantifying the osterix-positive osteoblast surfaces (vertebra and femur) and osteoid seams (vertebra), and changes were not observed in our flight mice. These results are inconsistent with reports indicating that bone formation was inhibited in rapidly growing rats^7–10^, which likely occurred because of bone growth inhibition. In astronauts, regardless of their level of physical exercise, an increase in serum or urine bone resorption markers is a consistent feature, whereas the level of bone formation markers are decreased^34,35^ or unchanged^36,37^. Furthermore, after one week of Earth recovery, we observed that active osteoclastic surfaces returned to the control value and that osteoblastic parameters increased in the trabecular compartments of both bone sites. Such bone formation rebound over the preflight values has also been observed in astronauts^34,35,38^ and may reflect the differentiation-related lag time of precursor cells in the bone response to mechanical 1 g reloading^39^. Overall, these data show that mature mice are a more appropriate human model than growing rats when investigating bone cellular alterations during and after spaceflight.

One-month spaceflight affects the vertebral column in a site-dependent manner. Micro-CT analyses revealed bone loss at the lumbar vertebral body, whereas the thoracic vertebral body was likely spared because of mechanical respiratory movements. Moreover, the BV/TV was initially higher in the thoracic vertebrae than the lumbar vertebrae (31 ± 1.5% vs. 17.9 ± 1.2%) and may have decreased the likelihood of spaceflight-induced bone loss in the thoracic vertebrae. In the caudal vertebrae, a site with no weight-bearing function, Berg-Johansen *et al*. also found a decrease in the BV/TV compared with that of the standard controls after the Bion-M1 mission^25^.

In the femur, the Flight animals suffered from more severe trabecular bone loss relative to the Habitat Control animals. This loss in the Flight group was associated with strut disappearance and disconnection, and thinning of the remaining struts. The Flight animals also displayed a thinner cortex compared with the Control animals as well as periosteal bone resorption and unaffected endosteal resorption compared with the Habitat Control group. Such cortical thinning has also been found in growing female mice after a shorter-duration spaceflight, and a further decrease in femur biomechanical properties (as assessed in the total femur by a three-point bending test) was reported^26^. In our study and concomitant with the in-flight bone structural alterations, we found that the hardness, or elastic modulus, and the maximal load measured by nanoindentation were deteriorated in the Flight animals at the periosteal side and the inner portion (the endosteal side was spared) of the lateral, anterior and posterior quadrants of the femoral cortex. At 8 days after re-entry, the femur showed no sign of trabecular recovery, a worsening of cortical thickness, and partial recovery of the nanomechanical parameters. Despite the decreased bone resorption and increased formation activities, no sign of bone structure rescue was observed.

To further understand the alteration of intrinsic local mechanical properties in-flight mice, we evaluated the bone mineral characteristics (i.e., DMB, crystallinity index, mineralization index, mineral maturity, and carbonation). No changes were evident, showing that the remaining bone preserved its mineral quality properties during spaceflight. We then evaluated the collagen quality because collagen is the main organic component of the bone matrix, and changes were not observed. Thus, to better comprehend the local impairment of mechanical properties, additional studies are still necessary. In particular, the orientation of the collagen fibers, which is known to be influenced by the loading direction^40^, should be further investigated, and the expression of enzymes involved in collagen crosslink formation should be assessed because an *in vitro* study showed that the conversion rate of immature crosslinks to mature compounds is decreased in microgravity^41^. Notably, recent investigations have shown that the nanomorphology and tissue mechanics are altered in mice subjected to osteocyte apoptosis induced by truncating connexin 43, which is the most abundant connexin protein in osteocyte gap junctions^42^.

By analysing more than 7000 osteocyte lacunae per femur cortex using SR imaging, we demonstrated that long-term spaceflight compromised the 3D lacunae shape descriptors. The most significant changes were a decrease in lacunar volume (with a greater proportion of small lacunae), with the lacunae becoming more spherical in the posterior zone, and the same trends were observed in the anterior, medial and lateral zones. Interestingly, the osteocyte lacunae in the calvaria (non-weight-bearing bone) have been shown to be more spherical that those observed in the fibula (a weight-bearing bone) in mature mice^43^. This finding suggested that osteocyte lacunae in weightless environments adapt their shape from elongated to more spherical, which is also observed in non-weight-bearing bones. A histological analysis of several hundred osteocytes per sample revealed that the fraction of empty lacunae was more than doubled in the Flight and Flight + Rec groups vs. the other groups, suggesting massive osteocyte death. More numerous empty osteocyte lacunae were also observed in an iliac crest biopsy of a Rhesus monkey flown on board the BION-11 flight^44^ and in neurectomised rat tibia by synchrotron imaging^45^. However, after a 14-day spaceflight, the empty lacunae ratio was unaffected and the 2D lacunae area increased in the pelvic bone of female mice^1^. It thus seems that different stages in osteocyte adaptation to spaceflight likely occur, and they are characterized by osteocyte osteolysis (MMP1a, 3, 10 and TracP5 overexpression)^1^ or osteocyte death as well as bone resorption stimulation. Osteocyte death also occurs after immobilization, microdamage and ageing^46^. Recently, osteocytic death during ageing has been considered to precede lacunae occlusion by minerals, which is called “micropetrosis”^47,48^. Therefore, in the Flight group, dead osteocytes may have left uninhabited lacunae that would progressively mineralize and shrink.

In conclusion, we provide evidence that a one-month spaceflight affects both axial and appendicular skeletal sites and leads to trabecular disconnection and cortical thinning, which are associated with increased osteoclastic resorption at the trabecular and periosteal levels, and fatty bone marrow. Although not exhaustive, multi-scale analyses of the bone matrix revealed new insights. At the tissue level, microradiography or FTIR measurements revealed that the bone mineral and collagen properties were preserved in the animals exposed to flight conditions. However, at the nanoscale level, the material properties were reduced at specific sites, such as at the trabeculae edges in the vertebrae or at the femur periosteal and mid-cortex. In the animals exposed to spaceflight conditions, osteocyte death was evidenced along with a smaller and more spherical shape of the osteocyte lacunae because of lacunar mineralization. Because osteocytes are long-lived key regulators of bone remodelling, their compromised survival may jeopardize bone deterioration reversibility, primarily in aging process. Further experiments should consider much longer post-flight periods to assess this hazard.

## Methods

The experimental design was approved by the IACUC of the MSU Institute of Mitoengineering (Protocol No. 35, 1 November, 2012) and the Biomedical Ethics Commission of the IBMP (Protocol No. 319, 4 April, 2013). This study in compliance with the European Convention for the Protection of Vertebrate Animals used for Experimental and Other Scientific Purposes^31^.

### Mice

Two groups of five C57BL/6N male mice exposed to one-month spaceflight on board the Russian Bion-M1 satellite were compared. One group was euthanized between 13–25 h after landing (Flight, n = 5), and the other group was euthanized 8 days later (Flight + Rec, n = 5). To evaluate the impact of spacecraft housing, two groups of ground control mice were investigated. A ground habitat control group (Habitat Control, n = 6) was placed in the same module under the same climate and food conditions as in the spacecraft, and a standard control group was placed in standard housing conditions (Control). For the microarchitecture, nanoindentation and FTIRM analyses, fifteen control mice were investigated. For the other investigations, 6 random control mice (always the same) were investigated. Three days prior to launch, all mice (except the Control group) were placed in the cylindrical animal module habitats (diameter, 98 mm; length, 200 mm; approximate volume, 1.7 cm^3^). Each habitat contained three mice. All groups were exposed to 12-h light/dark cycles with mean (±S.D.) temperatures of 23.0 ± 1.5 °C, 21.1 ± 0.4 °C and 21.3 ± 0.8 °C for the Habitat Control, Flight and Control groups, respectively. The Flight and Habitat Control animals consumed a paste-like diet containing 74.6% H_2_O and a casein gelling agent, whereas the Control mice consumed a standard pellet diet. The femurs and the vertebral columns were dissected and the soft tissue removed before being dehydrated for transport.

### Microtomography (µCT)

All left femurs were fixed in formalin and dehydrated in ethanol. All L3 and T12 vertebrae (n = 5/6) were scanned with a high-resolution μCT (VivaCT40, Scanco Medical, Bassersdorf, Switzerland) at the 12.5 μm cubic resolution and analysed as described in David *et al*.^49^. The following structural parameters of trabecular bone were generated: BV/TV (%), trabecular thickness (Tb.Th, mm), trabecular number (Tb.N, 1/mm), trabecular separation (Tb.Sp, mm), structure model index (SMI), connectivity density (Conn. D.), and degree of anisotropy (DA). The cortical thickness (mm), cortical bone area (mm^2^), and cortical tissue mineral density (TMD, gHA/cm^3^) were calculated by integrating the value on each transverse section. The cortical bone marrow area (mm^2^) was calculated as follows:

$${\rm{cortical}}\,{\rm{bone}}\,{\rm{marrow}}\,{\rm{area}}\,({{\rm{mm}}}^{2})={\rm{total}}\,{\rm{tissue}}\,{\rm{area}}\,({{\rm{mm}}}^{2})-{\rm{bone}}\,{\rm{area}}\,({{\rm{mm}}}^{2}).$$

For the protocol details, see the SI text.

### Histology and immunohistochemistry

Five and six left femurs and L3 vertebrae from the Flight and Flight + Rec and the Control and Habitat Control groups, respectively, were embedded in paraffin, cut and stained for osteoclast, osteoblast, osteocyte, marrow adiposity and growth plate assessments. For the protocol details, see the SI text.

### Fourier Transform Infrared spectroscopy

To assess the bone matrix characteristics, a different procedure was performed on femurs and vertebrae because a 2-µm thick section of femur could not be cut with a microtome (the mineral density was too high compared with that of the vertebrae). Thus, a macro-analysis (FTIR) was performed on the femur, and a microanalysis (FTIRM) was performed on the vertebrae. The FTIR analysis was performed on bone femur powder diluted in potassium bromide (KBr). Bone pellets were analysed in the transmission mode with a spectrum 100 spectrometer (Perkin-Elmer, Norwalk, CT, USA) equipped with a wideband detector (mercury–cadmium–telluride) (7800–400 cm^−1^). Twenty scans per spectrum were acquired at a 4 cm^−1^ resolution. The FTIRM analysis of the vertebrae was performed in the transmission mode on 2-μm thick sections with a Perkin-Elmer GXII Auto-image Microscope (Norwalk, CT, USA). Ten measurements per vertebrae were performed at a spatial resolution of 30 × 40 μm. Each spectrum was collected at a 4 cm^−1^ resolution, and 50 scans were performed per spectrum. For the protocol details, see the SI text. The following parameters were determined: mineral crystallinity index, which is inversely proportional to the full width at half maximum (FWMH) of the 604 cm^−1^ peak (apatitic phosphate environment) and corresponds to the crystal size and perfection (organization of the apatite lattice)^50^; the mineralization index, which is the area ratio of the bands of mineral matrix (ν_1_ν_3_PO_4_) over the organic matrix (Amide I) (1184–910 cm^−1^/1712–1592 cm^−1^), reflects the relative mineral content in the organic matrix; mineral maturity (calculated as the area ratio of the apatitic phosphate over nonapatitic phosphate (1030/1110 cm^−1^ area ratio)), corresponds to the progressive transformation of immature surface-hydrated domains into a mature and more stable apatite lattice and reflects the mineral age^50^; the collagen maturity, which is calculated as the ratio of the organic matrix bands in Amide I vibration (1660/1690 cm^−1^ area ratio) and reflects changes in the secondary structure of collagen in relation to the mineralization process^51,52^; and the carbonation, which is calculated as the area ratio of the ν_2_CO_3_ and ν_1_ν_3_PO_4_ bands and reflects the incorporation of CO_3_ ions in the crystal (including major site, type-B carbonate; minor site, type –A carbonate; and labile carbonate).

### Digitized microradiography

Five and six L2 vertebrae and cortical femur sections (approximately 150-µm thick) from the Flight and Flight + Rec and the Control and Habitat Control groups, respectively, were cut from the blocks embedded in methylmethacrylate with a precision diamond wire saw (Well, Escil, Chassieu, France). For the protocol details, see the SI text. For the cortical section, the following variables were measured using MATLAB software: mean DMB and heterogeneity index (HI = full width at half maximum of the curve of distribution of the mineralization). For the vertebrae, the HI measurements were not considered because of trabecular thickness heterogeneity. For the cortical analyses, three layers (endosteal, middle and periosteal zones) inside three anatomical regions (lateral, medial and anterior + posterior) were analysed independently because of possible mineralization heterogeneity between zones. The anterior and posterior zone analyses were pooled because the anterior and posterior zones could not be precisely differentiated in the femoral mid-diaphysis. The results are expressed in grams of mineral/cm^3^ of bone.

### Nanoindentation

All mid-diaphyses of the left femur and L1 vertebrae were embedded in polymethylmethacrylate and cut transversely to assess the bone material-level properties in the femur cortex and the vertebrae trabeculae. Because of potential differences in the bone material-level properties between the cortical zones, three anatomic cortical zones (anterior and posterior, medial, lateral) were analysed, thus allowing for measurements in areas with or without muscle fixation and with different mechanical loads. Within these anatomic zones, three zones of interest (endosteal, middle and periosteal) were analysed independently. Similarly, the interstitial trabecular vertebral bone (older formed bone) was analysed separately from the peripheral remodelled bone (younger formed bone). The following parameters were determined: maximal force (mN); hardness (mPa), which was interpreted as the mean pressure that the material can resist and calculated as the ratio of maximum force to contact area; modulus (gPa), which was defined by the initial slope of the unloading section of the curve; and dissipated energy (pJ), which was calculated as the area under the load-displacement curve. For the protocol details, see the SI text.

### Lacuna synchrotron radiation imaging and analysis

Five cortical femur sections per group were imaged using SR µCT on beamline ID19 at the ESRF (European Synchrotron Radiation Facility, Grenoble, France) with a 0.7 µm isotopic voxel size. For each sample, 2000 projection images were recorded over a total angle of 360° at a fixed energy of 26 keV. An approximately 2.7 × 2.7 × 1.5 mm^3^ field of view was scanned. The acquisitions were processed using a 3D-filtered back-projection algorithm to obtain 3D (2024)^3^ reconstructed images. To decrease the computational burden and independently analyse the anatomic specificity zones, four volumes of interest (VOIs) (medial, anterior, posterior and lateral) were determined within each volume at a size of 700 × 700 × 500 voxels, which corresponds to a physical size of 490 × 490 × 350 µm. The 3D images included several thousands of osteocyte lacunae per VOI. For the protocol details, see the SI text.

The total lacunar volume (Lc.V, mm^3^) was obtained directly by summing the individual volume of each lacuna. The lacunar number density (N.Lc/TV) and the lacunar volume density Lc.V/TV (%) were also assessed. Because the shape of the osteocyte lacunae is generally assumed to be ellipsoidal, second-order moments can efficiently be used to determine the lengths of the main axis of the best fitting ellipsoid. This method allowed for the extraction of the three-dimensional descriptor of lacunae: length (Lc.L_1_), width (Lc.L_2_) and depth (Lc.L_3_). The shape of the lacunae was evaluated by the ratios of the axis lengths Lc.L_1_/Lc.L_2_ and Lc.L_1_/Lc.L_3_. The canal volume fraction (Ca.V/TV, %) was calculated as (TV − (BV + Lc.V))/TV.

### Statistical analysis

A Kruskal Wallis test was performed on the four groups. When the Kruskal Wallis test was significant, a Mann-Whitney U test with Bonferroni correction (p critical value < 0.017) was performed between groups to test the effects of the spacecraft housing conditions, flight and recovery. The data are presented as clusters of individual points (bars = median); p < 0.017 was considered significant.

## Electronic supplementary material

Supplementary Info
