# Erratum: One-month spaceflight compromises the bone microstructure, tissue-level mechanical properties, osteocyte survival and lacunae volume in mature mice skeletons

**DOI:** 10.1038/s41598-017-09608-0

**Published:** 2017-10-12

**Authors:** Maude Gerbaix, Vasily Gnyubkin, Delphine Farlay, Cécile Olivier, Patrick Ammann, Guillaume Courbon, Norbert Laroche, Rachel Genthial, Hélène Follet, Françoise Peyrin, Boris Shenkman, Guillemette Gauquelin-Koch, Laurence Vico

**Affiliations:** 1 French National Centre for Space Studies, Paris, France; 2 INSERM, UMR 1059, University of Lyon, University Jean Monnet, F42023 S aint-Etienne, France; 3 INSERM, UMR 1033, University of Lyon, University Claude Bernard Lyon 1, F69622 Lyon, France; 4 University of Lyon, INSERM U1206, France and European Synchrotron Radiation Facility, CS40220, 38043, Grenoble, Cedex 9 France; 5 Division of Bone Diseases, Department of Internal Medicine Specialties, Geneva University Hospitals and Faculty of Medicine, Geneva, Switzerland; 6 CNRS UMR 5588, University of Grenoble Alpes, Grenoble, France; 7 Institute for Biomedical Problems, Russian Academy of Sciences, Moscow, Russia

*Scientific Reports* **7**:2659; doi:10.1038/s41598-017-03014-2; Article published online 01 June 2017

The original version of this Article contained errors in the spelling of the authors Maude Gerbaix, Vasily Gnyubkin, Delphine Farlay, Cécile Olivier, Patrick Ammann, Guillaume Courbon, Norbert Laroche, Rachel Genthial, Héléne Follet, Françoise Peyrin, Boris Shenkman, Guillemette Gauquelin-Koch, and Laurence Vico, which were incorrectly given as Gerbaix Maude, Gnyubkin Vasily, Farlay Delphine, Olivier Cecile, Ammann Patrick, Courbon Guillaume, Laroche Norbert, Gential Rachel, Follet Héléne, Peyrin Françoise, Shenkman Boris, Gauquelin-Koch Guillemette, and Vico Laurence.

These errors have now been corrected in the PDF and HTML versions of the Article.

