# Supplementary material for: One-month spaceflight compromises the bone microstructure, tissue-level mechanical properties, osteocyte survival and lacunae volume in mature mice skeletons
Source: Sci Rep. 2017 Jun 1;7:2659. doi: 10.1038/s41598-017-03014-2 (PMC5453937; doi:10.1038/s41598-017-03014-2)
Supplement: Supplementary file 1 — Supplementary Info [file 41598_2017_3014_MOESM1_ESM.pdf]

Supporting Information

Title:

One-month spaceflight compromises the bone microstructure, tissue-level mechanical properties, osteocyte survival and lacunae volume in mature mice skeletons

Short title: Effect of one-month spaceflight on the mouse skeleton

Authors: Gerbaix Maude[1, 2], Gnyubkin Vasily[2], Farlay Delphine[3], Olivier Cécile[4], Ammann Patrick[5], Courbon Guillaume[1], Laroche Norbert[2], Gential Rachel[6], Follet Hélène[3], Peyrin Françoise[4], Shenkman Boris[7], Gauquelin-Koch Guillemette[1], Vico Laurence[2]

[1] French National Centre for Space Studies, Paris, France

[2] INSERM, UMR 1059, University of Lyon, University Jean Monnet, F42023, Saint-Etienne, France

[3] INSERM, UMR 1033, University of Lyon, University Claude Bernard Lyon 1, F69622, Lyon, France

[4] University of Lyon, INSERM U1206, CNRS 5220, INSA Lyon, UCBL, CREATIS, 69621 Villeurbanne Cedex, France and European Synchrotron Radiation Facility, CS40220, 38043 Grenoble Cedex 9, France

[5] Division of Bone Diseases, Department of Internal Medicine Specialties, Geneva University Hospitals and Faculty of Medicine, Geneva, Switzerland

[6] CNRS UMR 5588, University of Grenoble Alpes, Grenoble, France

[7] Institute for Biomedical Problems, Russian Academy of Sciences, Moscow, Russia

Keywords: Spaceflight, Recovery, Bone structure, Bone quality properties, Mice

Correspondence to: vivo@univ-st-etienne.fr

SI Text

High resolution microtomography (µCT)

Data were acquired at 55 keV energy and 145 µA current for 12.5 µm cubic resolution. Right femur was scanned vertically under the distal growth plate until the end of the mid-diaphysis. Trabecular bone was analyzed in the secondary spongiosa within the metaphysis. Cortical bone was analyzed at the level of mid-diaphysis. Three-dimensional reconstructions were generated with the following parameters: Sigma: 1.5, Support: 2, Threshold: 260 (spongiosa) and Sigma: 0.8, Support: 1, Threshold: 260 (cortex). Three-dimensional structural parameters were obtained from 60 sections (0.75 mm) for trabecular and 30 sections (0.375 mm) for cortical bone. Entire L3 and T12 vertebrae were scanned horizontally. Trabecular bone was analyzed from a set of 37 sections between the two growth plates avoiding the primary spongiosa. Three-dimensional reconstructions were generated with the following parameters: Sigma: 1.2, Support: 2, and Threshold: 240. Trabecular regions of interest were manually defined by the same operator. Cortical contouring was automatically defined by Scanco software. The following structural parameters of trabecular bone: bone volume fraction (BV/TV, %), trabecular thickness (Tb.Th, mm), trabecular number (Tb.N, 1/mm), trabecular separation (Tb.Sp, mm), structure model index (SMI), connectivity density (Conn.D.), and degree of anisotropy (DA) were generated. Cortical thickness (mm), cortical bone area (mm^2), cortical tissue mineral density (TMD, gHA/cm^3) were calculated by integrating the value on each transverse section. Cortical bone marrow area (mm^2) was calculated as follows: cortical bone marrow area (mm^2) = total tissue area (mm^2) – bone area (mm^2).

Histology and immunohistochemistry

Femur and vertebrae were rehydrated for 12 h in 40% ethanol and kept at 4°C before being rinsed in isotonic solution (phosphate buffered saline solution). Then, samples were decalcified in immunocal formic acid decalcifier (Decal, Germany) for 12 h at 4°C before being washed in isotonic solution. To enable embedding, the samples were placed in embedding cassettes (Leica, Germany) and then dehydrated. Samples were placed for 9 h in each bath in a gradual series of dehydrating baths: 40% ethanol, 70% ethanol, 90% ethanol, 100% ethanol, and finally in two butanol baths. Paraffin embedding then took place using three sequential liquid paraffin baths of 9 h each, at 60°C and under vacuum conditions. 5-µm thick transversal sections of the diaphysis and longitudinal sections for femur metaphysis and vertebrae were cut with a 250-µm increment with a microtome (Leica RM2245, Wetzler, Germany). Prepared slides were stored at 4°C until required for staining. Immediately prior to staining, samples were placed in an incubator at 37°C for 30 min before being deparaffinized. Samples were deparaffinized and rehydrated using sequential baths for 5 min in 100% xylene, 100% xylene, then 100% ethanol , followed by 5 min in 100% ethanol, 96% ethanol, 70% ethanol , then 40% ethanol. Following deparaffinization, samples were stained according to the measurement being made and then mounted on a StarFrost adhesive glass slide.

To assess BV/TV, trabecular thickness, cortical area, cortical endosteal and periosteal perimeters, slices were stained with Sirius red solution (picric acid solution) for 10 min before washing in two 100% ethanol baths. Slides were dehydrated in methycyclohexane, then mounted.

To assess femur growth plate thickness and metaphysis marrow adiposity, slices were stained with eosin Y for 30 s, washed in demineralized water before haematoxcylin stain was added for 15 s before washing again with demineralized water. Slices were mounted using aqueous mounting solution.

To examine active osteoclast surfaces (Oc.S/BS, %), tartrate resistant acid phosphatase (TRAcP) staining was performed in vertebrae, femur metaphysis and cortical transversal sections. To examine osteoblast parameters (Ob.S/BS), osterix immunolabeling (primary antibody: polyclonal, rabit, 1/500, Ab22252, Abcam) and a biotinylated secondary goat anti-rabbit (Ab94361, rabbit specific HRP-AEC detection IHC kit) was used in vertebrae and in femur metaphysis. Only TRAcP positive cells osteoclast and Osterix positive osteoblast in contact with trabecula within the secondary spongiosa

or on cortical perimeters were included in analyses. To identify osteocyte nuclei which led to distinguish empty lacuna from full lacuna, cortical slices were stained with methyl green (M8884 Sigma-Aldrich). L1 vertebrae were embedded in methylmethacrylate, cut transversely and stained with Goldner's trichrome to assess osteoid surfaces (OS/BS, %). The aforementioned parameters of bone resorption and formation were measured at a magnification of x25 with a semiautomatic system consisting of a digitizing table (Summasketch-Summagraphics, Paris, France) connected to a personal computer and a Reichert Polyvar microscope equipped with a drawing system (Camera Lucida; Reichert-Jung Polyvar). To count the number of adipocytes and assess the growth plate thickness, a magnification of x10 was used using Bone Explora Nova software (version 3.50). The adipocyte cells were counted manually within the secondary spongiosa in the metaphysis of the femur. The total number of lacuna and the empty lacuna were counted manually in the endocortical zone (magnification of x20). All histology measurements were assessed by the same operator.

Fourier transform infrared spectroscopy

To assess bone matrix characteristics, a different procedure was performed on femur and vertebrae because it was not possible to cut a 2-µm thick section of femur with a microtome (mineral density too high compared to vertebrae). Thus, a macro-analysis (FTIR) was performed on femur and a microanalysis (FTIRM) was performed on vertebrae. FTIR analysis was performed on bone femur powder diluted in potassium bromide (KBr). Bone pellets were analyzed in transmission mode with a spectrum 100 spectrometer (Perkin-Elmer, Norwalk, CT, USA) equipped with a wideband detector (mercury–cadmium–telluride) (7800–400 cm^{-1}). Twenty scans by spectrum were acquired at 4 cm^{-1} resolution. On vertebrae, FTIRM analysis was performed in transmission mode on 2-µm thick sections with a Perkin-Elmer GXII Auto-image Microscope (Norwalk, CT, USA). Ten measurements per vertebrae were done, with a spatial resolution of 30 x 40 µm. Each spectrum was collected at 4 cm^{-1} resolution and 50 scans by spectrum. For protocol details, see the **SI** text. The following parameters were determined: mineral crystallinity index which is inversely proportional to the full width at half-maximum (FWHM) of the 604 cm^{-1} peak (apatitic phosphate environment) and corresponds to both crystal size and perfection (organization of the apatite lattice)[1]; mineralization index which is the area ratio of the bands of mineral matrix ($\nu_1\nu_3PO_4$) over organic matrix (Amide I) (1184–910 cm^{-1}/1712–1592 cm^{-1}), and reflects the relative content of mineral in organic matrix ; mineral maturity which is calculated as the area ratio of the apatitic phosphate over nonapatitic phosphate (1030/1110 cm^{-1} area ratio); this parameter corresponds to the progressive transformation of immature surface-hydrated domains into a mature and more stable apatite lattice and reflects the age of mineral[1]; collagen maturity which is calculated as the ratio of organic matrix bands in Amide I vibration (1660/1690 cm^{-1} area ratio) and reflects the change in secondary structure of collagen in relation to the mineralization process[2,3]; and carbonation which is calculated as the area ratio of the ν_2CO_3 and $\nu_1\nu_3PO_4$ bands and reflects the incorporation of CO_3 ions in the crystal (including major site, type-B carbonate; minor site, type –A carbonate and labile carbonate).

Digitized microradiography

Cross-sections were thinned between a plate and a frosted glass plate by silicon carbide grains (Escil, Chassieu, France) to a thickness of 50 ± 1 µm, were polished with a polisher (Escil, Chassieu, France) using 1 µm alumina suspension (Escil, Chassieu, France) and were cleaned in an ultrasonic device (Elma, Singen, Germany). The thickness was measured with a precision micrometric thickness comparator (precision of 1 µm, Compac, Geneva, Switzerland). The DMB was performed using a X-ray source L9421-02 (Microfocus Hammamatsu system) and a digitized detector (Photonic science CCD camera FDI VHR 11 M; active area: 36 × 24 mm (4008 × 2671 pixels), scintillator: Gd2O2S: Tb, aluminum filter: 12 µm) [4]. The exposure parameters were 40 kV, current of 50 µA and power of 2 W. The dynamic range of the detector was 12 bits (i.e., 4096 values). Each image was the average of 5 images (exposure time of 7 s each). An aluminum step-wedge reference (ultrapure aluminium, 99.5%, Strems Chemical Ltd, Strasbourg, France) with a known absorption coefficient was used (low

atomic number close to apatite). Greyness levels obtained at pixel level on bone sections were converted into DMB after plotting a calibration curve based on the values obtained from the aluminum standard.

Nanoindentation

Samples were rehydrated following a standardized protocol for 16 h in a saline solution before testing. A nanohardness tester (CSM Instruments, Peseux, Switzerland) was used to determine modulus and tissue hardness from a load-displacement curve obtained during indentation of a pyramidal diamond indenter as previously described [5]. Briefly, the indenter tip was loaded at a given depth into the sample and the load was then held constant, leading to a creeping of the material below the tip. Five indents per zones were set to a 900 nm depth with an approximate speed of 76 mN/min for all tested groups. Indents are always performed inside a lamella both in trabecular and cortical compartments and distal from osteocytes lacuna. At maximum load, a 5-s holding period was applied. The limit of the maximal allowable thermal drift was set to 0.1 nm/s.

Lacuna synchrotron radiation imaging and analysis

The first step consisted in bone segmentation to obtain bone volume (BV, mm^3) and total tissue volume (TV, mm^3) for each VOI. To provide binary imaging of bone volume and partially suppress noise within the reconstructed images, a Gaussian low pass filter and a simple thresholding was applied. Then, to obtain total volume (TV, mm^3), all cavities were filled. This preliminary investigation allowed evaluating the bone volume fraction (BV/TV, %) and total bone porosity (Total Po., %) evaluated as 100 minus BV/TV. Then, to segment the osteocyte lacunae within the region of the mask volume, a hysteresis thresholding with two thresholds set respectively to 15 and 127 was employed. The hysteresis thresholding requires to fix a low and a high threshold which were 15 and 127 in our case meaning that at the first step voxels with a grey value below 15 were assigned to background and higher than 127 were assigned to lacunae. Then the voxels with intermediate grey levels are iteratively assigned to either background or lacunae if they are connected to a background or lacunae voxel, leading to the final segmentation. As a result, a binary volume of osteocyte lacunae was acquired. The lacunae analysis was adapted from an efficient direct 3D analysis of osteocyte lacunae validated in human bone by Pei Dong et al [6]. Briefly, an automated quantification method has been designed to calculate the volume, length, width, height and anisotropy of each osteocyte lacuna. To quantify each individual cell, a connected component process was first performed to assign a label to each of them. The labelling was performed with a connectivity of 26 (meaning that each voxel was connected to its 26 3D neighbors). After labeling, all lacunae connected to the image borders were deliberately erased from the labeled volume to avoid the biased results that could be related to truncated lacunae. The total number of labels corresponds to the number of lacunae and is denoted N.Lc. By summing the number of voxels contained in each label, the volume of each lacuna was obtained. Even if hysteresis thresholding provided binary imaging, possible artifacts due to ring artifacts, physiological or nonphysiological microcracks or noise may have subsisted. These artifacts have different shape and size than that expected for lacunae. The objects smaller than 40 μm^3 were removed and considered as noise. The objects in which the volume was within the top 1% of the distribution were assumed to be artifactual lacunae, such as microcracks and canals, and were removed.

Table S1: Effects of Spaceflight, spacecraft housing and earth recovery on body masses and femur length

		Ctr		Habitat Ctr		Flight		Flight+Rec		KW Test	Mann-Whitney U Test				
	**Parameters**	**Mean**	**SE**	**Mean**	**SE**	**Mean**	**SE**	**Mean**	**SE**	**p**	**Ctr vs Habitat Ctr**	**Habitat Ctr vs Flight**	**Ctr vs Flight**	**Flight vs Flight+Rec**	**Flight+Rec vs Habitat Ctr**
**Total Body**	Launch body Mass (g)	26.05	0.41	27.63	0.51	26.96	0.89	27.68	1.09	0.208	0.037	0.662	0.349	0.691	1.000
	Landing body Mass (g)	27.71	0.56	27.85	0.59	29.30	1.11	25.06	1.05	0.073	0.910	0.662	0.266	0.032	0.052
	Delta body mass at landing (g)	1.66	0.26	0.22	0.74	2.34	1.29	-0.98	2.28	0.088	0.045	0.126	0.168	0.310	0.537
	Delta body mass after recovery (g)	1.66	0.26	0.22	0.74	2.34	1.29	-2.62	1.06	**0.005**	0.045	0.126	0.168	**0.016**	0.052
**Femur**	Femur length (mm)	16.52	0.20	16.23	0.25	16.62	0.25	16.11	0.20	0.305	0.394	0.329	1.000	0.111	0.476
	Growth plate thickness (µm)	216.9	5.2	167.0	9.6	144.2	19.4	132.7	6.7	**0.004**	**0.002**	0.247	0.030	1.000	0.017

Table S2: Effects of spaceflight, spacecraft housing and earth recovery on bone microarchitecture parameters

			Ctr		Habitat Ctr		Flight		Flight+Rec		KW Test	Mann-Whitney U Test				
	Parameters	Abbreviation	Mean	SE	Mean	SE	Mean	SE	Mean	SE	p	Ctr vs Habitat Ctr	Habitat Ctr vs Flight	Ctr vs Flight	Flight vs Flight+Rec	Flight+Rec vs Habitat Ctr
**Femur Trabecular**	Bone volume Fraction	BV/TV (%)	11.40	1.72	4.80	0.88	1.69	0.40	2.21	0.79	**0.000**	**0.014**	**0.009**	**0.000**	0.841	0.082
	Connectivity Density	Conn.D (1/mm^3)	53.2	10.9	16.6	6.0	1.8	0.6	4.0	2.8	**0.000**	0.023	**0.004**	**0.000**	1.000	0.052
	Structure model index	SMI	2.61	0.15	3.10	0.11	3.60	0.09	3.62	0.22	**0.000**	0.029	0.090	**0.000**	1.000	0.126
	Trabecular Number	Tb.N (1/mm)	3.88	0.17	3.04	0.20	2.56	0.16	2.80	0.39	**0.001**	**0.008**	0.082	**0.000**	0.690	0.537
	Trabecular thickness	Tb.Th (µm)	53.45	2.33	46.48	1.86	41.78	2.55	39.06	3.23	**0.001**	0.029	0.177	**0.011**	0.841	0.030
	Trabecular separation	Tb.Sp (mm)	0.263	0.011	0.347	0.022	0.404	0.023	0.383	0.046	**0.001**	**0.008**	0.082	**0.000**	1.000	0.537
	Tissue Mineral Density	TMD (mg HA/cm^3)	869	3	858	7	856	22	857	11	0.286	0.267	0.537	0.098	0.690	1.000
**Femur Cortical**	Cortical Area	Ct.Ar (mm^2)	0.92	0.02	0.88	0.02	0.83	0.03	0.74	0.03	**0.003**	0.267	0.117	0.066	0.056	**0.004**
	Marrow area	Ma.Ar (mm^2)	0.87	0.04	0.92	0.03	0.91	0.03	0.94	0.01	0.169	0.267	1.000	0.142	0.548	0.792
	Cortical Thickness	Ct.Th (mm)	0.215	0.003	0.205	0.003	0.194	0.005	0.175	0.005	**0.001**	0.112	0.052	**0.008**	0.032	**0.004**
	Cortical Bone Volume	Ct.BV (mm^3)	0.33	0.01	0.32	0.01	0.30	0.01	0.27	0.01	**0.003**	0.267	0.177	0.081	0.056	**0.004**
	Tissue Mineral Density	TMD (mg HA/cm^3)	1264	6	1264	2	1270	7	1267	8	0.900	0.850	0.537	0.672	1.000	0.429
**T 12 Vertebra Trabecular**	Bone volume Fraction	BV/TV (%)	31.00	1.46	28.50	0.64	22.99	1.91	27.19	2.46	0.072	0.310	0.520	0.017	0.222	0.931
	Connectivity Density	Conn.D (1/mm^3)	176.2	10.6	201.0	6.1	157.8	15.0	152.5	9.9	**0.024**	0.093	0.017	0.662	0.690	**0.009**
	Structure model index	SMI	0.56	0.14	0.89	0.09	1.36	0.17	0.76	0.25	**0.036**	0.132	0.082	**0.004**	0.151	0.537
	Trabecular Number	Tb.N (1/mm)	5.68	0.14	5.72	0.12	5.21	0.24	5.37	0.16	0.162	0.818	0.052	0.247	0.548	0.177
	Trabecular thickness	Tb.Th (µm)	58.30	1.40	54.77	0.66	52.12	1.26	56.20	2.30	**0.047**	0.065	0.126	**0.009**	0.222	0.329
	Trabecular separation	Tb.Sp (mm)	0.16	0.01	0.16	0.00	0.18	0.01	0.18	0.01	0.128	0.818	0.052	0.177	0.841	0.126
	Tissue Mineral Density	TMD (mg HA/cm^3)	804	4	803	2	803	12	821	4	**0.037**	0.699	0.429	1.000	0.151	**0.004**
**L3 Vertebra Trabecular**	Bone volume Fraction	BV/TV (%)	17.87	1.23	12.09	0.96	7.77	0.72	11.32	1.52	**0.000**	**0.002**	**0.009**	**0.000**	0.151	1.000
	Connectivity Density	Conn.D (1/mm^3)	147.5	8.9	101.2	10.5	37.0	12.1	79.4	22.9	**0.000**	**0.003**	**0.009**	**0.000**	0.095	2.470
	Structure model index	SMI	1.88	0.11	2.47	0.08	2.92	0.14	2.52	0.15	**0.000**	**0.002**	0.052	**0.000**	0.056	1.000
	Trabecular Number	Tb.N (1/mm)	5.77	0.12	5.38	0.17	5.07	0.23	4.90	0.12	**0.008**	0.112	0.177	0.033	0.841	0.082
	Trabecular thickness	Tb.Th (µm)	46.38	1.23	42.02	1.22	38.82	1.55	42.36	1.58	**0.015**	0.036	0.177	**0.005**	0.222	0.662
	Trabecular separation	Tb.Sp (mm)	0.17	0.00	0.19	0.01	0.20	0.01	0.21	0.01	**0.001**	0.066	0.126	**0.002**	1.000	0.126
	Tissue Mineral Density	TMD (mg HAcm^3)	723	5	705	7	727	8	733	7	0.094	0.066	0.082	0.800	0.421	0.030

Table S3: Effects of spaceflight, spacecraft housing and earth recovery on cellular parameters

			Ctr		Habitat Ctr		Flight		Flight+Rec		KW Test	Mann-Whitney U Test				
	**Parameters**	**Abbreviation**	**Mean**	**SE**	**Mean**	**SE**	**Mean**	**SE**	**Mean**	**SE**	**p**	**Ctr vs Habitat Ctr**	**Habitat Ctr vs Flight**	**Ctr vs Flight**	**Flight vs Flight+Rec**	**Flight+Rec vs Habitat Ctr**
**Femur trabecular**	Osteoblast surface/ Bone surface	Ob.S/BS (%)	3.79	0.53	2.12	0.40	1.48	0.48	3.20	0.70	**0.049**	0.056	0.310	0.032	0.095	0.222
	Osteoclast surface/ Bone surface	Oc.S/BS (%)	17.35	3.07	39.35	7.70	58.48	12.53	26.66	10.58	**0.029**	**0.015**	0.177	0.017	0.151	0.177
	Adipocytes density (1/mm^2)		0.45	0.07	0.36	0.11	7.81	2.48	7.10	2.30	**0.004**	0.310	**0.004**	**0.004**	0.310	0.082
**Femur Cortical**	Periosteal Osteoclast Surface/ Bone Surface	Ps Oc.S/BS (%)	8.65	1.78	14.06	5.23	32.45	5.34	9.82	2.45	**0.019**	0.690	0.032	**0.008**	**0.008**	0.690
	Endosteal Osteoclast Surface /Bone Surface	Ec Oc.S/BS (%)	0.00	0.00	4.12	0.48	0.77	0.77	1.59	1.08	**0.010**	**0.008**	0.032	0.690	0.690	0.151
	Total Lacuna Number	Total Lc.N	443.3	23.6	495.3	49.3	375.4	64.2	392.0	69.1	0.385	0.818	0.329	0.429	1.000	0.178
	Lacunar Density	Total Lc.N/BV (%)	0.09	0.01	0.10	0.01	0.07	0.01	0.09	0.01	0.422	0.394	0.126	0.662	0.421	0.662
	Empty Lacunar Fraction	Empty Lc.N/Total Lc.N (%)	4.59	0.96	3.64	0.58	11.44	1.50	9.71	1.68	**0.002**	0.485	**0.004**	**0.004**	0.310	**0.009**
**L3 Vertebra trabecular**	Osteoblast surface/ Bone surface	Ob.S/BS (%)	2.18	0.60	2.64	0.39	1.58	0.20	7.12	1.14	**0.010**	0.537	0.095	0.429	**0.008**	**0.016**
	Osteoclast surface/Bone surface	Oc.S/BS (%)	1.10	0.10	1.32	0.34	6.20	1.15	0.16	0.07	**0.001**	0.937	**0.009**	**0.004**	**0.008**	**0.004**
**L1 Vertebra Trabecular**	Osteoid Surface/Bone Surface	OS/BS (%)	2.01	0.18	1.50	0.14	1.57	0.21	2.90	0.42	**0.013**	0.082	1.000	0.151	**0.016**	**0.009**

Table S4: Effects of spaceflight, spacecraft housing and earth recovery on material quality properties measured by FTIR

			Ctr		Habitat Ctr		Flight		Flight+Rec		KW Test	Mann-Whitney U Test				
	Parameters	Abbreviation	Mean	SE	Mean	SE	Mean	SE	Mean	SE	P	Ctr vs Habitat Ctr	Habitat Ctr vs Flight	Ctr vs Flight	Flight vs Flight+Rec	Flight+Rec vs Habitat Ctr
**L2 Vertebra Trabecular**	Mineralization Index	IM	3.49	0.17	3.16	0.23	3.19	0.11	3.42	0.25	0.490	0.336	0.792	0.160	0.556	0.610
	Carbonation	CO3/PO4 ($\nu_2CO_3/\nu_1\nu_3PO_4$)	0.0063	0.000	0.0062	0.000	0.0057	0.000	0.0059	0.000	0.377	0.682	0.247	0.130	0.413	0.914
	Crystallinity	FWHM 604 cm^{-1}	30.51	0.47	30.08	0.82	29.50	0.90	29.52	0.44	0.579	0.750	0.662	0.328	1.000	0.610
	Mineral maturity	(1030 cm^{-1}/1110 cm^{-1})	0.65	0.09	0.70	0.11	0.58	0.12	0.72	0.13	0.868	0.750	0.662	0.646	0.556	1.000
	Collagen maturity	(1660 cm^{-1}/1690 cm^{-1})	1.5036	0.078	1.2146	0.083	1.2522	0.035	1.591	0.243	0.077	0.032	0.931	0.064	0.286	0.171
**Femur Cortical**	Mineralization Index	IM	1.8088	0.151	1.8883	0.269	1.854	0.268	2.16	0.248	0.705	1.000	1.000	0.724	0.691	0.429
	Carbonation	CO3/PO4 ($\nu_2CO_3/\nu_1\nu_3PO_4$)	0.0085	0.001	0.0089	0.002	0.0085	0.001	0.0064	0.001	0.085	0.228	0.792	0.724	0.056	0.082
	Crystallinity	FWHM 604 cm^{-1}	34.219	0.996	35.07	1.521	31.832	0.742	34.64	1.284	0.322	1.000	0.178	0.127	0.151	1.000
	Mineral maturity	(1030 cm^{-1}/1110 cm^{-1})	1.42	0.072	1.47	0.05	1.396	0.048	1.41	0.052	0.623	0.345	0.329	1.000	0.691	0.429
	Collagen maturity	(1660 cm^{-1}/1690 cm^{-1})	1.3625	0.059	1.38	0.082	1.332	0.046	1.228	0.153	0.956	0.755	1.000	0.943	0.691	0.792

Table S5: Effects of spaceflight, spacecraft housing and earth recovery on degree of mineralization measured by microradiography

			Ctr		Habitat Ctr		Flight		Flight+Rec		KW Test	Mann-Whitney U Test				
	Parameters	Abbreviation	Mean	SE	Mean	SE	Mean	SE	Mean	SE	p	Ctr vs Habitat Ctr	Habitat Ctr vs Flight	Ctr vs Flight	Flight vs Flight+Rec	Flight+Rec vs Habitat Ctr
**L2 Trabecular**	Total Degree of mineralization	DMB (g min/cm^3)	1,098	0,010	1,096	0,011	1,097	0,009	1,097	0,008	0,982	1,000	0,931	0,827	1,000	1,000
**Femur Cortical Total**	Total Degree of mineralization	DMB (g min/cm^3)	1,433	0,022	1,498	0,012	1,479	0,037	1,432	0,040	0,197	0,035	0,792	0,432	0,548	0,126
	Total Heterogenity Index	HI (g min/cm^3)	0,185	0,012	0,178	0,016	0,177	0,013	0,197	0,020	0,749	0,445	1,000	1,000	0,690	0,429
**Femur Cortical Lateral zone**	Periosteal DMB	DMB (g min/cm^3)	1,4029	0,023	1,4693	0,012	1,4639	0,035	1,404	0,052	0,097	0,022	0,792	0,202	0,222	0,126
	Periosteal HI	HI (g min/cm^3)	0,1975	0,019	0,1711	0,008	0,1891	0,014	0,176	0,016	0,746	0,445	0,537	1,000	0,691	1,000
	Middle DMB	DMB (g min/cm^3)	1,4651	0,025	1,553	0,017	1,5289	0,033	1,478	0,06	0,067	0,022	0,662	0,106	0,151	0,126
	Middle HI	HI (g min/cm^3)	0,1623	0,011	0,1745	0,01	0,1603	0,005	0,179	0,014	0,561	0,534	0,247	0,876	0,421	0,792
	Endosteal DMB	DMB (g min/cm^3)	1,4256	0,023	1,4921	0,021	1,4741	0,036	1,405	0,056	0,144	0,035	0,931	0,343	0,310	0,126
	Endosteal HI	HI (g min/cm^3)	0,1668	0,012	0,1527	0,006	0,1449	0,009	0,165	0,012	0,365	0,836	0,329	0,202	0,095	0,662
**Femur Cortical Medial zone**	Periosteal DMB	DMB (g min/cm^3)	1,4292	0,03	1,4818	0,014	1,4374	0,044	1,398	0,036	0,314	0,138	0,429	0,876	0,548	0,126
	Periosteal HI	HI (g min/cm^3)	0,1941	0,023	0,1443	0,01	0,1476	0,016	0,148	0,009	0,153	0,073	0,931	0,106	0,691	1,000
	Middle DMB	DMB (g min/cm^3)	1,4328	0,021	1,5199	0,017	1,4961	0,054	1,469	0,039	0,252	**0,014**	0,792	0,530	0,841	0,537
	Middle HI	HI (g min/cm^3)	0,1505	0,006	0,1402	0,008	0,1324	0,003	0,137	0,008	0,323	0,445	0,662	0,073	1,000	0,792
	Endosteal DMB	DMB (g min/cm^3)	1,3927	0,028	1,4692	0,017	1,445	0,035	1,406	0,03	0,256	0,073	0,931	0,530	0,548	0,082
	Endosteal HI	HI	0,1384	0,01	0,127	0,003	0,1295	0,008	0,148	0,008	0,392	0,945	0,931	1,000	0,056	0,082
**Femur Cortical Anterior and posterior zone**	Periosteal DMB	DMB (g min/cm^3)	1,4122	0,02	1,4633	0,011	1,4635	0,034	1,415	0,036	0,175	0,035	0,792	0,268	0,421	0,126
	Periosteal HI	HI (g min/cm^3)	0,1945	0,024	0,1824	0,013	0,1631	0,009	0,209	0,022	0,517	1,000	0,329	0,432	0,151	0,662
	Middle DMB	DMB (g min/cm^3)	1,4661	0,025	1,5388	0,012	1,5168	0,036	1,468	0,042	0,209	0,051	1,000	0,268	0,548	0,126
	Middle HI	HI (g min/cm^3)	0,1622	0,009	0,1507	0,01	0,139	0,008	0,153	0,01	0,430	0,534	0,429	0,106	0,421	1,000
	Endosteal DMB	DMB (g min/cm^3)	1,4049	0,022	1,4587	0,016	1,4529	0,043	1,402	0,04	0,288	0,073	0,792	0,432	0,548	0,178
	Endosteal HI	HI (g min/cm^3)	0,1788	0,01	0,1931	0,022	0,1782	0,012	0,175	0,012	0,976	1,000	1,000	1,000	0,691	0,792

Table S6: Effects of spaceflight, spacecraft housing and earth recovery on biomechanical properties assessed by nano indentation

			Ctr		Habitat Ctr		Flight		Flight+Rec		KW Test	Mann-Whitney U Test				
	Zones	Parameters	Mean	SE	Mean	SE	Mean	SE	Mean	SE	p	Ctr vs Habitat Ctr	Habitat Ctr vs Flight	Ctr vs Flight	Flight vs Flight+Rec	Flight+Rec vs Habitat Ctr
L1 Vertebra Trabecular	Interstitial bone	Maximal Force (mN)	8.50	0.39	8.66	0.64	8.08	0.63	9.18	0.59	0.551	0.622	0.662	0.673	0.095	0.537
		Modulus (gPa)	11.41	0.50	12.35	0.52	11.20	0.64	11.35	0.78	0.507	0.267	0.178	0.866	1.000	0.247
		Hardness (mPa)	322.4	16.9	329.2	28.2	302.5	26.8	364.5	30.7	0.457	0.910	0.537	0.800	0.151	0.429
		Dissipated Energy (pJ)	2887	133	3014	195	2832	209	3034	147	0.738	0.519	0.537	0.933	0.691	1.000
	Peripheral bone	Maximal Force (mN)	5.95	0.26	6.20	0.39	5.92	0.34	6.53	0.42	0.643	0.677	0.931	1.000	0.421	0.429
		Modulus (gPa)	7.90	0.34	8.74	0.35	6.81	0.26	7.78	0.52	**0.042**	0.080	**0.009**	0.098	0.222	0.126
		Hardness (mPa)	211.9	11.2	212.3	17.0	202.3	11.1	239.1	17.2	0.548	0.791	0.662	1.000	0.310	0.429
		Dissipated Energy (pJ)	2291	82	2492	123	2449	168	2455	139	0.469	0.267	0.931	0.395	1.000	0.792
Femur Cortical Lateral zone	Periosteal zone	Maximal Force (mN)	9.65	0.41	10.17	0.97	6.85	0.26	8.93	0.69	**0.017**	0.622	0.030	**0.001**	0.032	0.329
		Modulus (gPa)	16.08	0.50	16.19	0.68	12.88	0.64	14.82	1.06	**0.027**	0.733	0.030	**0.002**	0.151	0.247
		Hardness (mPa)	444.3	24.6	477.0	52.7	291.8	15.9	412.5	44.7	**0.015**	0.470	0.017	**0.001**	0.056	0.429
		Dissipated Energy (pJ)	2978	82	3055	244	2359	76	2680	83	**0.007**	0.569	0.082	**0.000**	0.056	0.429
	Middle zone	Maximal Force (mN)	14.99	0.59	15.04	0.79	11.63	0.90	12.59	1.45	0.056	1.000	0.017	**0.015**	1.000	0.177
		Modulus (gPa)	21.38	0.52	20.29	1.04	18.70	0.31	19.55	1.48	0.071	0.424	0.177	**0.008**	0.222	0.662
		Hardness (mPa)	746.9	36.5	756.8	39.5	563.8	50.5	609.7	85.5	**0.045**	0.850	0.017	**0.015**	1.000	0.247
		Dissipated Energy (pJ)	4189	139	4237	248	3217	269	3489	271	**0.020**	0.791	0.030	**0.011**	0.548	0.126
	Endosteal zone	Maximal Force (mN)	15.29	0.54	16.01	1.11	13.21	1.81	13.00	1.84	0.480	0.424	0.329	0.395	0.841	0.247
		Modulus (gPa)	20.40	0.39	20.26	0.90	19.33	1.43	19.19	1.70	0.976	0.910	0.931	0.735	0.841	1.000
		Hardness (mPa)	772.4	33.2	820.9	65.7	662.2	110	628.8	107.2	0.403	0.424	0.329	0.395	1.000	0.177
		Dissipated Energy (pJ)	4246	162	4474	262	3607	390	3721	406	0.138	0.470	0.082	0.119	1.000	0.177
Femur Cortical Medial zone	Periosteal zone	Maximal Force (mN)	11.53	1.18	11.88	1.55	9.16	1.00	9.99	0.74	0.518	0.602	0.247	0.343	0.841	0.429
		Modulus (gPa)	17.07	0.82	16.79	1.07	16.24	0.83	14.90	2.26	0.986	0.968	0.792	1.000	0.841	0.792
		Hardness (mPa)	568.3	69.7	597.4	97.6	425.8	54.5	489.6	40.1	0.505	0.602	0.247	0.343	0.421	0.429
		Dissipated Energy (pJ)	3303	276	3209	307	2621	249	2692	198	0.406	0.904	0.247	0.186	0.841	0.429
	Middle zone	Maximal Force (mN)	14.22	0.82	13.67	0.67	11.70	1.42	12.98	0.99	0.461	0.602	0.329	0.186	0.548	0.792
		Modulus (gPa)	21.02	0.73	19.85	0.22	19.06	1.53	19.00	2.31	0.516	0.239	0.662	0.298	0.841	0.662
		Hardness (mPa)	707.0	47.2	682.6	38.9	557.1	73.2	656.8	53.3	0.314	0.659	0.177	0.107	0.310	0.662
		Dissipated Energy (pJ)	3911	226	3634	218	3224	396	3412	277	0.380	0.353	0.662	0.186	0.841	0.662
	Endosteal zone	Maximal Force (mN)	12.29	0.84	12.73	0.90	11.85	1.14	12.00	1.03	0.984	1.000	0.662	0.893	1.000	0.931
		Modulus (gPa)	18.31	0.68	18.61	0.79	18.38	0.71	17.86	1.72	0.970	0.602	0.792	0.964	1.000	0.662
		Hardness (mPa)	594.9	49.3	627.2	57.3	576.5	68.5	589.9	58.7	0.983	0.841	0.662	1.000	0.841	0.931
		Dissipated Energy (pJ)	3553	206	3464	233	3190	375	3248	207	0.626	0.602	0.329	0.391	0.690	0.662
Femur	Periosteal	Maximal Force (mN)	12.08	0.86	12.54	0.54	9.36	0.67	11.56	1.72	0.300	0.910	0.017	0.142	0.548	0.662
		Modulus (gPa)	17.48	0.76	17.18	0.40	16.18	1.08	17.35	0.93	0.788	0.622	0.662	0.445	0.421	1.000

**Cortical Anterior and posterior zone**	zone	Hardness (mPa)	596.3	51.5	630.0	32.1	440.4	34.8	562.6	103.0	0.260	0.850	**0.009**	0.142	0.548	0.662
		Dissipated Energy (pJ)	3432	190	3441	143	2661	115	3290	376	0.108	0.910	**0.009**	0.033	0.222	0.792
	Middle zone	Maximal Force (mN)	15.19	0.75	14.77	0.80	12.43	0.40	14.08	1.02	0.327	0.677	0.052	0.197	0.310	0.537
		Modulus (gPa)	21.35	0.72	20.20	0.54	20.14	0.78	20.83	0.27	0.264	0.205	0.662	0.197	0.222	0.247
		Hardness (mPa)	755.9	44.3	748.6	49.6	597.3	16.8	696.8	62.9	0.293	0.910	0.017	0.197	0.548	0.329
		Dissipated Energy (pJ)	4229	204	4071	210	3308	161	3847	279	0.062	0.677	0.030	0.015	0.151	0.537
	Endosteal zone	Maximal Force (mN)	13.32	0.59	12.79	0.30	11.97	1.16	13.02	1.47	0.615	0.569	0.537	0.230	0.548	0.662
		Modulus (gPa)	18.74	0.51	18.09	0.31	18.64	0.78	18.84	0.58	0.545	0.132	0.792	0.800	0.690	0.429
		Hardness (mPa)	654.9	33.7	634.7	20.2	576.8	66.2	648.6	92.4	0.620	0.622	0.429	0.266	0.548	0.662
		Dissipated Energy (pJ)	3844	175	3570	99	3367	340	3708	386	0.493	0.304	0.662	0.197	0.310	0.792

Table S7: Effect of spaceflight, housing spacecraft and recovery on femur cortical osteocytes lacuna characterization by 3D synchrotron imaging

			Ctr		Habitat Ctr		Flight		Flight+Rec		KW Test	Mann-Whitney U Test				
	Parameters	Abbreviation	Mean	SE	Mean	SE	Mean	SE	Mean	SE	p	Ctr vs Habitat Ctr	Habitat Ctr vs Flight	Ctr vs Flight	Flight vs Flight+Rec	Flight+Rec vs Habitat Ctr
**Anterior zone**	Lacuna Number	N.Lc	1809	278	2056	216	1672	121	1575	159	0.353	0.691	0.286	0.730	0.686	0.111
	Lacuna Volume	Lc.V (mm^3)	5.4E-04	1.E-04	5.9E-04	6.E-05	4.5E-04	4.E-05	3.9E-04	6.E-05	0.154	0.691	0.111	0.413	0.486	0.064
	Total Porosity	Total Po. (%)	1.768	0.342	1.770	0.123	1.488	0.126	1.608	0.222	0.520	1.000	0.111	0.413	1.000	0.556
	Lacuna vol. Fraction	Lc.V/TV (%)	0.013	0.002	0.014	0.001	0.012	0.001	0.012	0.001	0.332	0.841	0.111	0.413	0.886	0.111
	Canal vol. Fraction	Ca.V/TV (%)	1.755	0.340	1.756	0.123	1.474	0.125	1.597	0.221	0.521	1.000	0.111	0.413	1.000	0.556
	Lacuna density	N.Lc/TV ($1/mm^3$)	43968	5937	49654	3688	44687	3860	47516	3379	0.773	0.691	0.413	0.730	0.486	0.905
	Lacuna mean volume	mean Lc.V (μm^3)	294.9	15.3	289.9	18.6	272.1	12.4	245.8	14.2	0.176	1.000	0.413	0.556	0.343	0.111
	Lacuna mean L1/L2	mean Lc.L1/L2	1.96	0.10	1.89	0.03	1.88	0.05	1.95	0.03	0.546	0.421	1.000	0.413	0.343	0.413
	Lacuna mean L1/L3	mean Lc.L1/L3	5.55	0.27	4.96	0.24	5.56	0.10	5.40	0.24	0.414	0.151	0.191	1.000	0.886	0.556
**Lateral zone**	Lacuna Number	N.Lc	2113	230	1874	266	1650	306	1678	218	0.608	0.556	0.691	0.556	1.000	0.691
	Lacuna Volume	Lc.V (mm^3)	6.2E-04	9.E-05	5.9E-04	8.E-05	4.7E-04	9.E-05	4.3E-04	5.E-05	0.259	0.905	0.421	0.286	0.548	0.222
	Total Porosity	Total Po. (%)	3.968	0.527	3.578	0.489	3.318	0.569	2.928	0.500	0.580	0.905	1.000	0.556	0.691	0.421
	Lacuna vol. Fraction	Lc.V/TV (%)	0.018	0.002	0.375	0.363	0.012	0.002	0.016	0.002	0.397	0.286	0.691	0.111	0.421	0.691
	Canal vol. Fraction	Ca.V/TV (%)	3.951	0.524	3.200	0.407	3.306	0.567	2.910	0.497	0.557	0.413	0.841	0.556	0.691	0.841
	Lacuna density	N.Lc/TV ($1/mm^3$)	63463	4843	43929	5067	44326	7156	61145	9269	0.119	0.064	0.841	0.191	0.222	0.095
	Lacuna mean volume	mean Lc.V (μm^3)	286.9	13.5	316.5	11.3	287.0	15.7	258.1	14.6	0.133	0.286	0.310	0.905	0.151	0.056
	Lacuna mean L1/L2	mean Lc.L1/L2	1.95	0.03	2.02	0.04	1.96	0.02	1.91	0.03	0.271	0.413	0.310	1.000	0.310	0.095
	Lacuna mean L1/L3	mean Lc.L1/L3	3.80	0.10	4.09	0.24	4.11	0.17	3.54	0.18	0.120	0.191	1.000	0.286	0.056	0.151
**Medial zone**	Lacuna Number	N.Lc	1393	138	1361	129	971	186	1299	196	0.205	1.000	0.056	0.111	0.571	0.786
	Lacuna Volume	Lc.V (mm^3)	5.0E-04	4.E-05	4.8E-04	4.E-05	2.9E-04	4.E-05	3.3E-04	8.E-05	**0.037**	0.730	0.032	**0.016**	1.000	0.143
	Total Porosity	Total Po. (%)	1.863	0.147	1.882	0.208	1.396	0.246	1.213	0.278	0.203	0.730	0.222	0.191	1.000	0.143
	Lacuna vol. Fraction	Lc.V/TV (%)	0.014	0.000	0.013	0.001	0.009	0.001	0.011	0.002	**0.039**	0.413	**0.016**	**0.016**	0.786	0.393
	Canal vol. Fraction	Ca.V/TV (%)	1.848	0.147	1.867	0.208	1.389	0.245	1.202	0.277	0.203	0.730	0.222	0.191	1.000	0.143
	Lacuna density	N.Lc/TV ($1/mm^3$)	34686	7266	37272	2871	26993	6097	42111	6359	0.318	1.000	0.151	0.413	0.251	0.571
	Lacuna mean volume	mean Lc.V (μm^3)	362.5	23.9	353.3	21.5	310.1	25.7	254.2	43.6	0.146	0.730	0.548	0.413	0.393	0.071

	Lacuna mean L1/L2	mean Lc.L1/L2	1.87	0.04	1.83	0.01	1.88	0.02	1.91	0.05	0.206	0.286	0.016	0.730	1.000	0.393
	Lacuna mean L1/L3	mean Lc.L1/L3	5.36	0.28	5.16	0.22	5.30	0.20	5.48	0.36	0.938	0.730	0.691	1.000	0.786	1.000
**Posterior zone**	Lacuna Number	N.Lc	1763	96	1577	133	1393	163	1446	137	0.348	0.393	0.556	0.111	1.000	0.413
	Lacuna Volume	Lc.V (mm^3)	3.8E-04	2.E-05	3.9E-04	4.E-05	2.9E-04	5.E-05	2.5E-04	3.E-05	0.055	1.000	0.191	**0.016**	0.486	0.032
	Total Porosity	Total Po. (%)	1.470	0.246	1.704	0.172	1.333	0.169	1.055	0.088	0.081	0.571	0.286	0.191	0.210	**0.016**
	Lacuna vol. Fraction	Lc.V/TV (%)	0.010	0.001	0.011	0.001	0.010	0.001	0.009	0.001	0.321	0.393	0.730	**0.016**	0.486	0.064
	Canal vol. Fraction	Ca.V/TV (%)	1.459	0.249	1.694	0.171	1.326	0.168	1.045	0.088	0.081	0.571	0.286	0.191	0.210	**0.016**
	Lacuna density	N.Lc/TV ($1/mm^3$)	49338	4104	46278	2845	47798	5012	49416	3886	0.924	0.786	0.730	0.413	0.686	0.730
	Lacuna mean volume	mean Lc.V (μm^3)	216.5	9.3	244.5	7.5	205.7	13.9	173.9	6.6	**0.012**	0.143	**0.016**	0.413	0.210	**0.016**
	Lacuna mean L1/L2	mean Lc.L1/L2	1.855	0.014	1.889	0.038	1.744	0.034	1.868	0.029	**0.046**	0.786	**0.016**	0.730	0.057	1.000
	Lacuna mean L1/L3	mean Lc.L1/L3	5.851	0.162	5.414	0.159	5.318	0.236	5.839	0.187	0.186	0.143	0.730	1.000	0.210	0.286

1. Farlay, D., Panczer, G., Rey, C., Delmas, P.D. & Boivin, G. Mineral maturity and crystallinity index are distinct characteristics of bone mineral. *J Bone Miner Metab* 28, 433-445 (2010).

2. Paschalis, E.P., *et al.* Spectroscopic characterization of collagen cross-links in bone. *J Bone Miner Res* 16, 1821-1828 (2001).

3. Farlay, D., *et al.* The ratio 1660/1690 cm(-1) measured by infrared microspectroscopy is not specific of enzymatic collagen cross-links in bone tissue. *PLoS One* 6, e28736 (2011).

4. Montagner, F., *et al.* Validation of a novel microradiography device for characterization of bone mineralization. *J Xray Sci Technol* 23, 201-211 (2015).

5. Brennan-Speranza, T.C., Rizzoli, R., Kream, B.E., Rosen, C. & Ammann, P. Selective osteoblast overexpression of IGF-I in mice prevents low protein-induced deterioration of bone strength and material level properties. *Bone* 49, 1073-1079 (2011).

6. Dong, P., *et al.* 3D osteocyte lacunar morphometric properties and distributions in human femoral cortical bone using synchrotron radiation micro-CT images. *Bone* 60, 172-185 (2014).
